# Amelogenesis Imperfecta; Genes, Proteins, and Pathways

**DOI:** 10.3389/fphys.2017.00435

**Published:** 2017-06-26

**Authors:** Claire E. L. Smith, James A. Poulter, Agne Antanaviciute, Jennifer Kirkham, Steven J. Brookes, Chris F. Inglehearn, Alan J. Mighell

**Affiliations:** ^1^Division of Oral Biology, School of Dentistry, St. James's University Hospital, University of LeedsLeeds, United Kingdom; ^2^Section of Ophthalmology and Neuroscience, St. James's University Hospital, University of LeedsLeeds, United Kingdom; ^3^Section of Genetics, School of Medicine, St. James's University Hospital, University of LeedsLeeds, United Kingdom; ^4^Oral Medicine, School of Dentistry, University of LeedsLeeds, United Kingdom

**Keywords:** amelogenesis, amelogenesis imperfecta, ameloblasts, enamel, biomineralization, Leiden Open Variant Database, LOVD, amelogenesis genetics

## Abstract

Amelogenesis imperfecta (AI) is the name given to a heterogeneous group of conditions characterized by inherited developmental enamel defects. AI enamel is abnormally thin, soft, fragile, pitted and/or badly discolored, with poor function and aesthetics, causing patients problems such as early tooth loss, severe embarrassment, eating difficulties, and pain. It was first described separately from diseases of dentine nearly 80 years ago, but the underlying genetic and mechanistic basis of the condition is only now coming to light. Mutations in the gene *AMELX*, encoding an extracellular matrix protein secreted by ameloblasts during enamel formation, were first identified as a cause of AI in 1991. Since then, mutations in at least eighteen genes have been shown to cause AI presenting in isolation of other health problems, with many more implicated in syndromic AI. Some of the encoded proteins have well documented roles in amelogenesis, acting as enamel matrix proteins or the proteases that degrade them, cell adhesion molecules or regulators of calcium homeostasis. However, for others, function is less clear and further research is needed to understand the pathways and processes essential for the development of healthy enamel. Here, we review the genes and mutations underlying AI presenting in isolation of other health problems, the proteins they encode and knowledge of their roles in amelogenesis, combining evidence from human phenotypes, inheritance patterns, mouse models, and *in vitro* studies. An LOVD resource (http://dna2.leeds.ac.uk/LOVD/) containing all published gene mutations for AI presenting in isolation of other health problems is described. We use this resource to identify trends in the genes and mutations reported to cause AI in the 270 families for which molecular diagnoses have been reported by 23rd May 2017. Finally we discuss the potential value of the translation of AI genetics to clinical care with improved patient pathways and speculate on the possibility of novel treatments and prevention strategies for AI.

## Introduction

**Figure 1:**
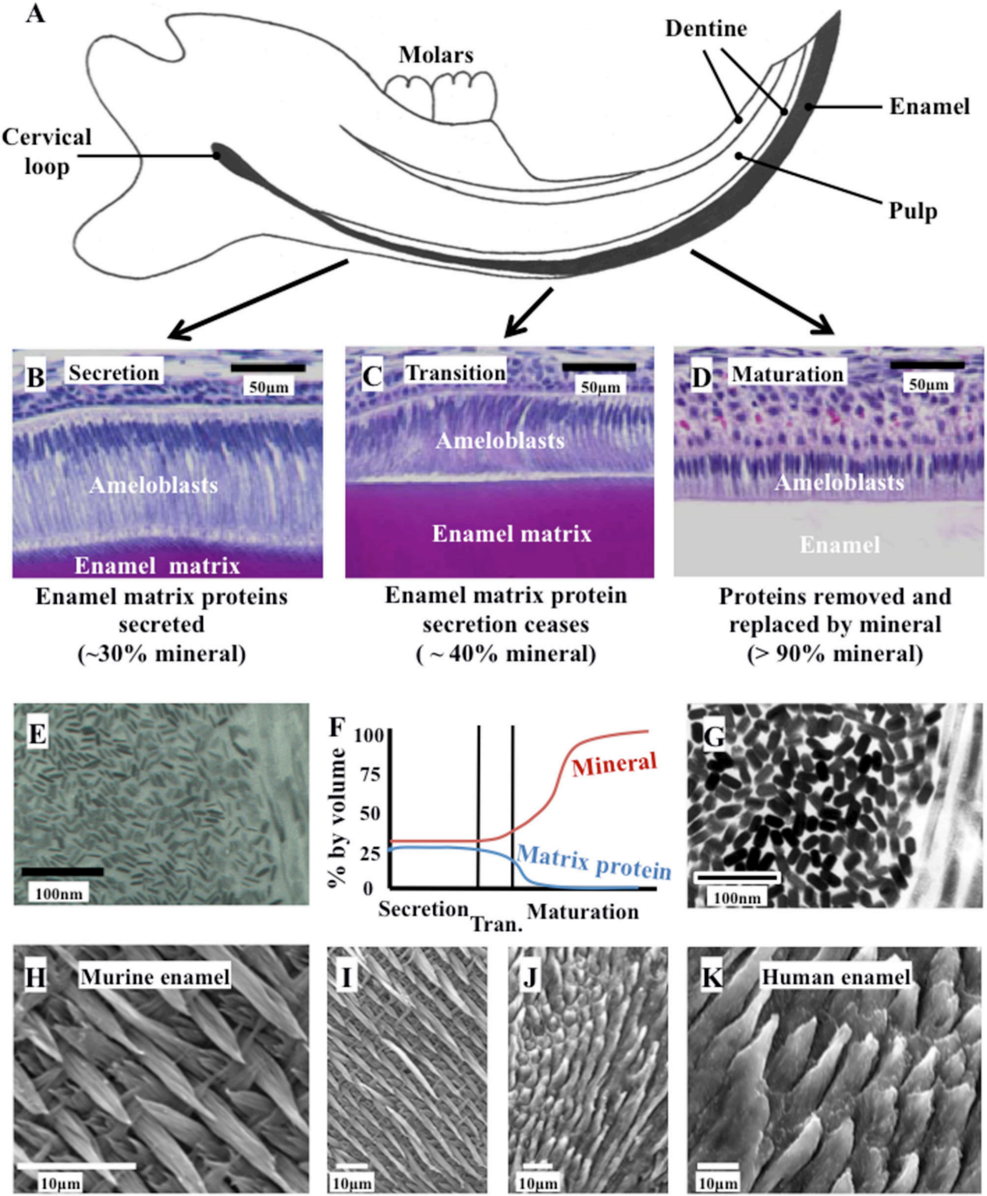
Ameloblast morphology, crystal development, and the final structure of the enamel. **(A)** Schematic cross section of the murine incisor. **(B–D)** Histology of the murine incisor. **(B)** During secretion, ameloblasts exhibit an elongated morphology with a cellular extension (the Tomes' process); **(C)** during transition the Tomes' process degenerates and the ameloblasts begin to reduce in height; **(D)** during maturation, the ameloblasts remove nearly all protein from the developing enamel and supply mineral ions to support crystallite growth; **(E)** immature enamel crystalites form during secretion by growth in their long axis; **(F)** by the end of secretion, the developing enamel is around 30% mineral and 25% matrix protein, with the remainder tissue fluid. By the end of maturation, the enamel is nearly 100% mineral; **(G)** the enamel crystallites grow in width and thickness during enamel maturation; **(H)** and **(I)** murine enamel has a decussating arrangement of enamel prisms; **(J)** and **(K)** human enamel is also arranged in a prismatic structure. Elements from this figure have been adapted from previously published figures and we acknowledge the following publications and publishers for the elements specified: Panels **(A–D)** were previously published by Barron et al. (2010). Panel **(E)** was previously published by Robinson (2014).

Mature enamel is the hardest, most mineralized tissue in the human body, comprising >95% by weight crystals of substituted calcium hydroxyapatite (HA; Ca_10_[PO_4_]_6_[OH]_2_). Enamel consists of a highly organized structure of interwoven prisms and inter-prismatic material, both made up of HA crystals (Figure 1). This structural organization and chemical composition provide the mechanical strength to withstand long-term use. The enamel-forming cells, the ameloblasts, are lost upon tooth eruption. Consequently enamel lacks any capacity for cellular repair and once formed, must function over a lifetime.

### Amelogenesis

Amelogenesis is the process of enamel formation. It takes place in three, well-defined stages known as the secretory, transition and maturation phases (Figure 1). The initial differentiation, positioning and orientation of ameloblasts, as well as their coordinated functioning as a cohort, are also crucial to amelogenesis.

**Figure 2:**
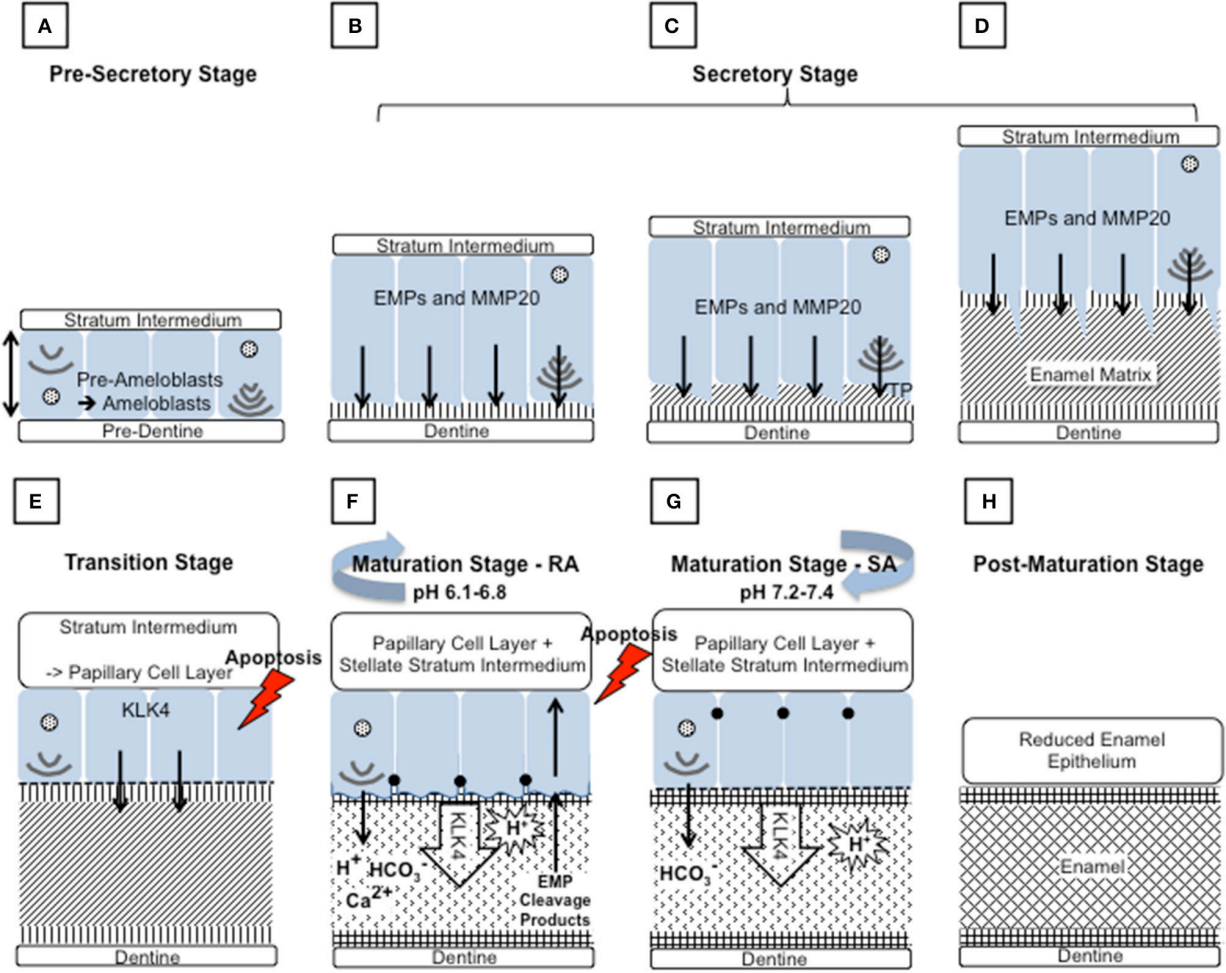
Schematic diagram depicting the main events during amelogenesis. **(A)** Pre-secretory stage: Ameloblasts (blue) differentiate from the cells of the inner enamel epithelium (IEE), in response to reciprocal signaling between the IEE and the dental papilla. The basal lamina between the IEE and dental papilla breaks down so that the cells are in contact with the pre-dentine. The ameloblasts elongate and their nuclei shift to the proximal side of the cell, nearest the stratum intermedium (SI), resulting in reversal of the ameloblasts' polarity. At the distal end, closest to the pre-dentine, the Golgi apparatus and rough endoplasmic reticulum increase in size to increase the capacity for protein production, post translational modification and secretion. The non-dividing cell becomes further polarized as it forms a distal extension that will go on to form the Tomes' process (TP). Each ameloblast develops and maintains anchoring junctions to hold the ameloblast layer in alignment and to control what passes between them. **(B–D)** Secretory stage: During the secretory stage, a proteinaceous extracellular matrix is secreted from the ameloblast TP, as the ameloblast layer retreats from the dentine layer. To achieve this, ameloblasts produce large amounts of membrane bound, secretory granules containing enamel matrix proteins (EMPs). EMPs are constitutively secreted via exocytosis into the extracellular space at the distal end of the cell, on to the newly formed dentine. **(B)** Mineral immediately forms in this initial enamel matrix and forms a close association with the dentine mineral. This will form the aprismatic enamel. **(C)** The ameloblasts begin to move away from the dentine and further develop their TP at the distal end. EMPs are secreted from two aspects of the ameloblasts to produce enamel matrix that will go on to form the prismatic and interprismatic enamel. **(D)** As secretion progresses the TP lengthens and thins. The portion secreting the prismatic enamel is reduced before secretion ceases, therefore the final enamel formed will be aprismatic. **(E)** Transition stage: The transition stage is characterized by reduced EMP secretion and internal reorganization of the ameloblasts. Ameloblasts shorten to around half their original height and reduce in volume. Their nuclei become more central and the ER is reduced in size. The TP is completely lost and an atypical basal lamina is formed against the enamel matrix. Ameloblasts adhere to the enamel matrix via hemidesmosomes. The cells of the SI, stellate reticulum and the outer enamel epithelium form the papillary layer (PL). Capillaries invaginate into this layer and overlay the ameloblasts. The cells of the PL may assist ameloblasts in the maturation stage by participating in ion transport and removal of enamel protein products and water from the developing enamel. The ameloblast population reduces by around 25% at this stage through apoptosis. **(F)** and **(G)** Maturation stage: During the maturation stage the partially mineralized enamel matrix becomes fully mineralized by the breakdown and removal of residual EMPs, and the growth in width and thickness of enamel crystallites. These processes are achieved through repeated cyclical processes. The ameloblasts act as a gated barrier for the movement of ions and degraded proteins between the SI and the developing enamel and vice versa. To achieve this, the ameloblast membrane facing the enamel matrix modulates between ruffle ended **(F)** and smooth ended **(G)** morphologies. This is achieved in coordinated groups of ameloblasts across the developing enamel. Ruffle ended ameloblasts (RA) form membrane invaginations and tight junctions at the apical end, near the enamel surface, whereas smooth ended ameloblasts (SA) are more leaky. Enamel crystal growth generates large amounts of protons but it has also been shown that protons are pumped into the enamel by RA. Both RA and SA release bicarbonate ions into the enamel that act as a buffer to increase pH. A mildy acidic pH is found in enamel at RA regions and a more neutral pH in SA regions. During maturation around 25% of ameloblasts apoptose. **(H)** Post-maturation stage: The ameloblasts and other cells of the enamel organ, form the reduced enamel epithelium, which eventually contributes to the junctional epithelium of mature teeth. However, many of the ameloblasts apoptose before the formation of the junction epithelium is completed.

Amelogenesis involves the secretion of a proteinaceous matrix in which immature enamel HA crystallites are deposited. The matrix is then degraded and concurrently replaced, almost entirely, with HA mineral (Figure 2). During the secretary phase, the ameloblasts move away from the dentino-enamel junction (DEJ), secreting a soft extracellular protein matrix by exocytosis from cellular extensions (known as Tomes' processes) to fill the space they leave behind (Skobe, 1976). During the transition stage, which begins as the matrix achieves the thickness of the future enamel, matrix protein secretion decreases and the ameloblasts restructure (Reith, 1970). During the maturation stage, the matrix proteins are degraded by proteases and replaced with tissue fluid. Maturation stage ameloblasts increase their active transport of mineral ions into the fluid, which drives the growth of the pre-existing enamel crystallites in width and thickness (Robinson et al., 1995). During this stage, ameloblasts alternate between a ruffle ended and smooth ended morphology in groups of coordinated cells (Warshawsky and Smith, 1974). These different morphologies reflect cyclical changes in ameloblast function related to the regulation of pH and the control of ion transport so that the enamel becomes progressively more mineralized until the crystallites occlude the tissue volume. The matrix is transformed into mature enamel that is almost devoid of protein (Smith, 1998).

Throughout the transition and maturation stages, around 50% of the ameloblasts undergo apoptosis (Smith and Warshawsky, 1977). Post maturation, the surviving ameloblasts either apoptose or go on to contribute to the junctional epithelium of mature teeth (Bosshardt and Lang, 2005).

### Amelogenesis imperfecta

#### Definition and phenotypes

**Figure 3:**
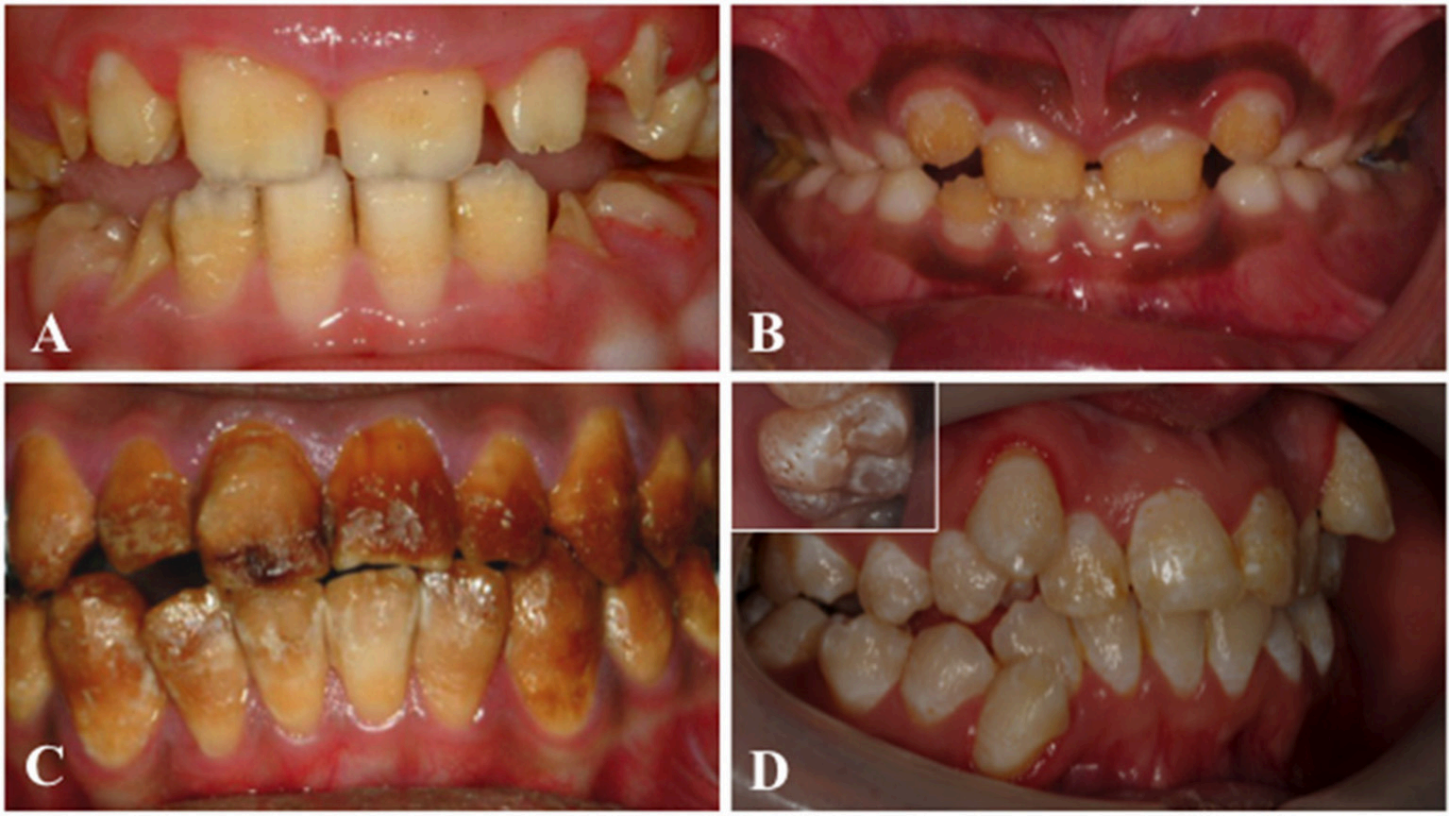
Clinical images that illustrate the variability of AI. **(A)** Hypoplastic AI is characterized by teeth without the curves associated with a normal enamel volume. **(B)** In hypomaturation AI enamel volume can be near-normal, but opaque with structural weaknesses that result in rapid post-eruptive enamel loss with enamel fracturing away to exposure the underlying dentine. **(C)** Brown discolouration and early post-eruptive enamel loss is typical of hypomineralised forms of AI. **(D)** Mixed AI phenotypes are frequently encountered. In this example a near-normal enamel volume is characterized by multiple focal pits that are most evident on the inset image, with variable colouration that includes focal opacities, but without premature fracturing of the enamel to reveal dentine.

Amelogenesis imperfecta (AI) is a heterogeneous group of genetic conditions characterized by defects in the formation of enamel in all teeth of both dentitions. In an effort to classify the disease, particular phenotypes have been defined but this approach can be confounded by mixed phenotypes (Figure 3). *Hypoplastic AI* describes thin but mineralized enamel, or in extreme cases, the complete absence of enamel, that results from failure during the secretory stage. *Hypomineralized AI* is caused by maturation stage failure, giving rise to enamel that is of full thickness but is weak and fails prematurely. The hypomineralized phenotype can be further subdivided into *hypomaturation* and *hypocalcified AI*. The former is caused by incomplete removal of protein from the enamel matrix and produces brittle enamel, while the latter is characterized by insufficient transport of calcium ions (Ca^2+^) into the developing enamel and produces soft enamel.

Phenotyping of teeth from AI patients is complicated by post-eruptive changes that occur during the time spent in the mouth. Obtaining unerupted genotyped human embryonic teeth would be difficult and ethically questionable, while the study of erupted teeth precludes the direct study of amelogenesis (though enamel composition and ultrastructure can provide some form of record of the events occurring during amelogenesis). Therefore, mouse models have proved invaluable to AI research. Murine phenotyping of AI disease models can utilize the continuously erupting incisor to view all stages of amelogenesis at once, or gain a snapshot of amelogenesis via analysis of embryonic/neonatal unerupted molar teeth. Figure 1 shows the histology of the murine incisor and Supplementary Table 1 summarizes a selection of the AI-relevant mouse models that have been well characterized to date. The table also highlights genes for which murine models have not yet been described or for which an abnormal dental phenotype has not been reported.

#### Prevalence, impact, and treatment

AI is reported to range in frequency in different populations from 1 in 700 to 1 in 14,000 (Witkop and Sauk, 1976; Backman and Holm, 1986; Crawford et al., 2007), and has a significant impact upon patients and healthcare provision (Coffield et al., 2005). AI enamel is abnormally thin, soft, fragile, pitted and/or discolored, causing patients severe embarrassment, eating difficulties and pain. It is also associated with negative social outcomes and poor aesthetics (Hashem et al., 2013). AI is very difficult to treat and there is a weak evidence base to inform clinical decision-making and management choices (Dashash et al., 2013). Interventions focus on aesthetics and maintaining occlusal height and tooth function, whilst maintaining the natural dentition for as long as possible. Diagnostically it is necessary to distinguish AI from more common enamel defects such as fluorosis, molar incisor hypomineralization (Gotler and Ratson, 2010) and those caused by time-limited events, such as serious systemic illness (Salanitri and Seow, 2013).

## The genetics of amelogenesis imperfecta

AI was first described as a separate clinical entity to dentinogenesis imperfecta in 1938 (Finn, 1938) and its study has helped to define the processes and genes involved in amelogenesis. Since the discovery, over 25 years ago, that mutations in amelogenin, X linked (*AMELX*) lead to AI (Lagerstrom et al., 1991), many other genes have been shown to be defective in AI. The falling costs of next generation sequencing (NGS) have accelerated the identification of new genes for AI. These discoveries have also expanded the known functions of proteins mutated in AI from solely the structural enamel matrix proteins and their proteolytic processing enzymes, to a range of other proteins, involved in diverse functions, such as vesicle transport, pH sensing and cell adhesion.

Here we review advances in our understanding of the molecular basis of AI presenting in isolation of other health problems with recognition that co-segregating health problems may present later in life. For some of the genes included, other mutations can cause more widespread health problems beyond AI. We focus on the more recently identified AI genes, but include all reported genes, reviewing the functions of the proteins that they encode, the mutations identified and the resulting enamel phenotypes. We also summarize the murine models available and document any enamel phenotypes observed in these. Furthermore, we highlight a new online resource (http://dna2.leeds.ac.uk/LOVD/), detailing nearly two hundred published AI-causing mutations identified in two hundred and seventy families reported by 23rd May 2017. This resource represents an open repository for all interested in advancing the understanding of amelogenesis. We conclude by considering how these advances might impact on clinical care in future.

### The enamel matrix proteins

The first AI-causing mutations were identified in the genes encoding the enamel matrix proteins (EMPs), known to make up the bulk of the secreted enamel organic matrix. The EMP genes evolved from a common ancestral gene (Sire et al., 2007) and form part of the secretory calcium-binding phosphoprotein gene cluster. Their encoded proteins have a distinctive architecture of a signal peptide and a conserved casein kinase 2 phosphorylation domain likely to be targeted by family with sequence similarity 20, member C (FAM20C, MIM ^*^611061) (Yang et al., 2016).

The enamel matrix proteins include amelogenin (AMELX, MIM ^*^300391), which makes up around 90% of the EMPs secreted by ameloblasts (Termine et al., 1980), with the remaining 10% comprising ameloblastin (AMBN, MIM ^*^610259) and enamelin (ENAM MIM ^*^606585), in order of abundance (Smith, 1998). The alternative splicing and extracellular proteolytic processing of amelogenin, ameloblastin and enamelin have been reviewed elsewhere (Brookes et al., 1995; Iwata et al., 2007; Kobayashi et al., 2007; Moradian-Oldak, 2012). However, it is becoming increasingly clear that it is not only perturbation of the proteins' extracellular roles as part of the enamel matrix that is important in AI, but also the proteins' aberrant intracellular processing and the outcome of the unfolded protein response (UPR; Brookes et al., 2014).

#### AMELX

Amelogenin is a hydrophobic, proline and histidine rich protein, thought to act as an enamel matrix pH buffer (Guo et al., 2015) and as a scaffold for the spacing and growth of enamel crystallites (Chen et al., 2011). It is subject to extensive extracellular proteolytic processing following its secretion (Brookes et al., 1995). It is regarded as a tooth specific protein since it has not been detected elsewhere in human tissues (Chan et al., 2011). However, in murine tissues, it has been detected in dentine-forming, cementum-forming and bone-forming cells, as well as in the developing eye and brain (Fong and Hammarstrom, 2000; Janones et al., 2005; Haze et al., 2007).

*AMELX* mutations cause X-linked AI (MIM #301200) (Lagerstrom et al., 1991). Heterozygous mutations tend to present in female AI patients as stripes of normal and AI affected enamel due to lyonization (Berkman and Singer, 1971). In males, a copy of *AMELX* exists as *AMELY* on the Y chromosome, but *AMELY* transcription is around 10% of that of *AMELX* (Aldred et al., 1992; Salido et al., 1992) and hence cannot compensate for loss of *AMELX* expression. The AI phenotype in males is determined by the type and position of the mutation. Large deletions and N-terminal variants cause a hypomaturation AI defect with variable focal hypoplasia while mutations in the signal peptide and toward the C terminus cause smooth hypoplastic AI (Hart et al., 2002).

Over twenty *AMELX* mutations have been reported, including large deletions, frameshifts, nonsense and missense variants. For the majority of variants, pathology is thought to be due to loss of function, though an *Amelx* mutation in mice has been linked to toxic gain of function via activation of the pro-apoptotic UPR (Brookes et al., 2014). Altered splicing, due to variants such as the silent c.120T>C (NM_182680.1) change, has also been shown to result in enamel pathology (Cho et al., 2014). This particular variant prevents the excision of exon 4 from the majority of *AMELX* transcripts, thus preventing the formation of a miRNA from the normally excised exon 4 (Le et al., 2016). These examples show that further study, within the context of *AMELX* alternative splicing and its roles in signaling, is required to accurately define disease mechanisms.

#### ENAM

Enamelin, the largest of the EMPs, is a tooth specific acidic protein expressed primarily by secretory stage ameloblasts (Hu and Yamakoshi, 2003). It is successively cleaved from its C terminus, resulting in numerous products (Fukae et al., 1993; Hu and Yamakoshi, 2003; Lu et al., 2008). Like amelogenin and ameloblastin, the uncleaved protein is found only within the newly secreted, outermost layer of the enamel matrix and is thought to be involved in enamel crystal extension (Hu et al., 2008). Some ENAM cleavage products, such as the 32 kDa fragment identified in pigs, have high affinity for HA crystals and accumulate within and between the enamel prisms (Hu and Yamakoshi, 2003).

The first mutation identified in *ENAM* caused an autosomal dominant AI with a severe, smooth hypoplastic phenotype (MIM #104500) as a result of a dominant-negative effect of aberrant splicing (Rajpar et al., 2001) and a milder, local hypoplastic phenotype (MIM #204650) caused by missense mutations (Mardh et al., 2002). Autosomal recessive inheritance has also been documented for *ENAM* mutations (Hart et al., 2003; Ozdemir et al., 2005a; Chan et al., 2010); homozygotes and heterozygotes present with a severe and a milder, local form, respectively (Ozdemir et al., 2005a). Severity based on zygosity is more often seen with nonsense or frameshift variants that escape nonsense mediated decay (NMD).

#### AMBN

AMBN is rich in glycine, leucine and proline and, in addition to within the enamel matrix, localizes to the Tomes' processes and the DEJ (Krebsbach et al., 1996; MacDougall et al., 2000), but has also been detected in pre-odontoblasts, developing tooth roots and craniofacial bone (Fong et al., 1996, 1998; Spahr et al., 2006). AMBN is expressed throughout amelogenesis (Lee et al., 1996) but peaks during the secretory stage (Fukumoto et al., 2004). *AMBN* transcripts undergo alternative splicing to form two isoforms (Krebsbach et al., 1996; Hu et al., 1997). Porcine ameloblastin is extensively modified and is cleaved upon secretion by MMP20 to form a number of protein products that accumulate within different compartments of the enamel matrix. For example, N-terminal products accumulate between the enamel prisms throughout the matrix (Bartlett and Simmer, 1999). Although, it is known that AMBN can influence the differentiation and proliferation of ameloblasts (Fukumoto et al., 2004), it is also important in extracellular signaling to induce osteoblast differentiation (Iizuka et al., 2011), cell adhesion, via heparin and fibronectin (Beyeler et al., 2010), and mineralization (Yamakoshi et al., 2001; Zhang et al., 2011).

Only two mutations have been reported in *AMBN* in AI patients, both discovered through NGS. The first *AMBN* mutation reported, a large, in-frame deletion encompassing exon 6, segregated with recessive hypoplastic AI (MIM #616270) in a consanguineous Costa Rican family (Poulter et al., 2014c). Scanning electron microscopy showed both reduced mineral density and enamel thickness, mirroring the murine *Ambn*^−5, 6/−5, 6^ model. The second homozygous mutation, thought to alter splicing, was identified in one patient in a large cohort with oro-dental disease, using a targeted NGS assay (Prasad et al., 2016a).

### The enamel matrix proteases

Another group of genes for which a candidate approach identified AI-causing mutations are those encoding the enamel matrix proteases. These enzymes include matrix metallopeptidase 20 (MMP20, MIM ^*^604629), which specifically cleaves the enamel matrix proteins during the secretory stage to produce functional peptides, and kallikrein related peptidase 4 (KLK4, MIM ^*^603767) that proteolytically degrades the enamel matrix proteins to facilitate their removal by endocytosis during the maturation stage.

#### MMP20

Matrix metallopeptidases (MMPs) influence cell motility by regulating cell interactions and matrix degradation, crucial processes in many aspects of development (VanSaun and Matrisian, 2006). Like other MMPs, MMP20 is a zinc dependent endopeptidase that is secreted in an inactive precursor form that requires cleavage for its activation (Llano et al., 1997). MMP20 is secreted by ameloblasts concurrent with the EMPs, and is responsible for cleavage of EMP at specific residues, shortly after their secretion (Simmer and Hu, 2002). This generates products with specific, diverse roles during amelogenesis. MMP20 has been shown to be necessary for controlling HA crystal morphology (Prajapati et al., 2016) and through its action on amelogenin, may regulate mineralization (Kwak et al., 2016). MMP20 is also capable of cleaving the extracellular domains of cadherins that mediate cell-cell interactions as part of adherens junctions to allow ameloblast cell movement (Guan and Bartlett, 2013; Guan et al., 2016). This may affect amelogenesis since ameloblasts must move in synchronous groups in order to form typical enamel architecture. Since cadherins are linked to the actin cytoskeleton via catenins, cadherin cleavage releases β-catenin, which can act as a transcription factor and may be important for ameloblast differentiation (Bartlett et al., 2011; Guan et al., 2016).

Mutations in *MMP20* lead to autosomal recessive hypomaturation AI (MIM #612529) (Kim et al., 2005). Eleven missense, nonsense, frameshift and splice site mutations have been described, all resulting in a similar phenotype (Ozdemir et al., 2005b; Papagerakis et al., 2008; Lee et al., 2010a; Gasse et al., 2013; Wang et al., 2013b). All five of the missense variants reported lie within either the catalytic peptidase domain or the hemopexin domain, which, through homology, is thought to influence substrate specificity or to bind inhibitors or activators of the pro-enzyme.

#### KLK4

*KLK4* encodes a serine protease that is expressed and secreted by ameloblasts in both the transition and maturation stages of amelogenesis (Hu et al., 2000, 2002; Simmer et al., 2009). Like MMP20, newly secreted KLK4 must be cleaved for its activation. *In vitro* experiments have shown that MMP20 can activate newly secreted KLK4 and that KLK4 can inactivate MMP20, potentially explaining the shift in proteinase activity during the transition stage (Yamakoshi et al., 2013).

KLK4 acts to further degrade the enamel proteins already cleaved by MMP20 during secretion and is capable of functioning over the wide pH range that occurs during maturation (Smith, 1998; Bartlett, 2013). Such activity aids removal of protein from the developing enamel by maturation stage ameloblasts, allowing the enamel crystallites to grow in width and thickness (Simmer et al., 2009; Bartlett, 2013).

*KLK4* mutations cause autosomal recessive hypomaturation AI (Hart et al., 2004). All four *KLK4* variants reported so far are either nonsense or frameshift mutations (Hart et al., 2004; Wright et al., 2011; Wang et al., 2013b; Smith et al., 2017a). However, only two of the four are predicted to lead to NMD. Of the two frameshift mutations affecting codons in the final exon, and therefore not expected to undergo NMD, one alters one of the three catalytic residues, p.S207, essential to the function of all kallikrein enzymes. This mutation has been shown to result in greatly reduced protein expression and proteolytic function (Seymen et al., 2015). Prior to the identification of the frameshift variant, c.632delT only three *KLK4* variants in four families had been identified. However, the c.632delT variant, reported to occur at a frequency of 0.15% in the South Asian population, has been reported in five Pakistani families with hypomaturation AI and is predicted to disrupt three of six structurally important disulphide bonds (Smith et al., 2017a). Characterization of the human enamel phenotype revealed that overall the enamel was hypomineralized but that the deeper (inner) enamel was more seriously affected than the more superficial (outer) enamel (Smith et al., 2017a).

### Cell-cell and cell-matrix adhesion

For amelogenesis to proceed, the ameloblasts must function as a coordinated cohort and must maintain their contact with the secreted extracellular matrix (ECM) not only as they retreat from the dentine surface during secretion, but also during transition and maturation. These contacts require specific molecules, including integrins, laminins and collagens, and structures such as desmosomes and hemidesmosomes. The basal lamina degrades as the pre-ameloblasts undergo their terminal differentiation and an atypical basal lamina is then reformed during the transition stage. During the secretory stage, the Tomes' processes act as the contact point between the ameloblast and the enamel matrix.

#### ITGB6

Integrin, β6 (ITGB6; MIM ^*^147558) is a member of a large family of cell surface-adhesion receptors that mediate cell-cell and cell-ECM interactions by facilitating interaction with the cytoskeleton (Alberts et al., 2002). ITGB6 is predominantly found in epithelial cells and forms a heterodimer with integrin subunit alpha V (Busk et al., 1992; Breuss et al., 1993). Within the developing tooth, ITGB6 localizes predominantly in maturation stage ameloblasts (Wang et al., 2014b).

ITGB6 is known to bind to arginine-glycine-aspartic acid (RGD) motifs which are found in ECM proteins such as fibronectin, as well as the latency associated peptide of transforming growth factor-β1 (TGF-β1) (Breuss et al., 1993; Weinacker et al., 1994; Munger et al., 1999; Annes et al., 2002). Via this interaction and other mechanisms, ITGB6 is able to activate TGF-β1 (Munger et al., 1999).

An *Itgb6* null mouse exhibited a hypomineralized AI phenotype, with the loss of any ordered enamel prism arrangement (Mohazab et al., 2013). Accumulation of amelogenin in the enamel matrix and the presence of enamel pits were also noted (Mohazab et al., 2013). Patients with mutations in *ITGB6* have since been reported (MIM #616221) with either hypomineralized pitted enamel, similar to that reported in the *Itgb6* null mouse (Poulter et al., 2014a), or hypoplastic enamel with a rough surface (Wang et al., 2014b), both recessively inherited. More recently, Ansar et al. (2015) reported a consanguineous family with a homozygous *ITGB6* mutation with adolescent alopecia, intellectual disability and dentogingival abnormalities with rough, discolored enamel. However, it is unclear if these additional phenotypes result from the *ITGB6* variant or are co-segregating, for example, due to an undetected copy number variant. The *ITGB6* missense mutations identified so far lie within the β1 domain of the protein involved in binding to α integrin subunits, activity-modifying cations and ligands (Xiong et al., 2001, 2002). Wang et al. (2014b) also reported a patient with a homozygous *ITGB6* nonsense mutation, but phenotyping of the enamel was complicated by the co-presence of a hemizygous Nance-Horan syndrome (congenital cataracts and dental anomalies) mutation (MIM ^*^300457).

#### LAMA3, LAMB3, and COL17A1

Laminin, alpha 3 (*LAMA3*; MIM ^*^600805), laminin, beta 3 (*LAMB3*; MIM ^*^150310) and laminin gamma 2 (*LAMC2*; MIM ^*^150292) encode the three subunits of the heterotrimeric protein laminin 332 (LM332), which localizes to epithelial basement membranes of the ECM (Aberdam et al., 1994a). LM332 has a central role in the assembly and stability of hemidesmosomes, structures that mediate attachment between cells and the ECM (Nievers et al., 1999). Collagen type XVII, alpha-1 (COL17A1; MIM ^*^113811) is a constituent of hemidesmosomes and is a ligand for LM332 (Nishie et al., 2011; Van den Bergh et al., 2011). Mutations in the genes encoding COL17A1 or the subunits of LM332 cause the autosomal recessive condition junctional epidermolysis bullosa (JEB; MIM #226700, #226650), in which failure to form hemidesmosomes between skin layers leads to extensive skin blistering (Aberdam et al., 1994b; Pulkkinen et al., 1994a,b; Kivirikko et al., 1995; McGrath et al., 1995). In addition, JEB patients often present with hypoplastic, pitted enamel (Wright et al., 1993), and it has been noted that heterozygous carriers of some mutations in these genes sometimes have AI in the absence of any skin phenotype (McGrath et al., 1996; Yuen et al., 2012; Kim et al., 2013; Poulter et al., 2014b).

LM332 has been implicated in ameloblast adhesion to the enamel surface via interaction with integrin alpha 6 beta 4 within hemidesmosomes; and in cell migration via binding of integrin alpha 3 beta 1 (Carter et al., 1991; Marchisio et al., 1993). Mature ameloblasts and Tomes' processes show particularly strong staining for mature LM332 protein (Yoshiba et al., 1998), which is thought to participate in the control of ameloblast differentiation and adhesion to the enamel matrix. This is supported by analysis of tooth buds from JEB patients, which show ameloblast disorganization and subsequent reduction in enamel volume (Brain and Wigglesworth, 1968). Enamel in JEB patients also has a number of changes in chemical composition, suggesting that mineral transport or ameloblast metabolism is affected (Kirkham et al., 2000).

No enamel phenotype has been reported for *Lamb3* null mice since they die in early post-natal life (Kuster et al., 1997). In contrast, for *Lama3* null mice, ameloblasts were smaller than those in wild-type (WT) mice, suggesting that LM332 is required for normal ameloblast differentiation (Ryan et al., 1999). Enamel deposition was described as abnormal and the enamel epithelium was disorganized (Ryan et al., 1999).

COL17A1 is expressed throughout enamel formation (Asaka et al., 2009). *Col17*^−/−^ mice have fewer hemidesmosomes than WT mice and exhibit thin, disorganized Tomes' processes (Asaka et al., 2009). This may be the result of alterations in ameloblast differentiation due to lack of contact with, and signals from, mesenchymal tissues. Mineralization is delayed in *Col17*^−/−^ mice. The enamel formed lacks the typical regular prism structure and the prisms themselves are malformed, a phenotype reminiscent of the *Lama3*^−/−^ mouse but not as severe (Asaka et al., 2009). Enamel proteins such as AMELX, AMBN, and ENAM are expressed by ameloblasts at a significantly lower level in *Col17*^−/−^ mice than WT, whereas expression of the pre-secretory protein tuftelin is increased, again suggesting that ameloblast differentiation is incomplete in *Col17*^−/−^ mice (Asaka et al., 2009).

Heterozygous carriers of some *LAMA3, LAMB3*, and *COL17A1* mutations present with hypoplastic AI (MIM #104530) (Murrell et al., 2007; Pasmooij et al., 2007; Yuen et al., 2012; Kim et al., 2013; Poulter et al., 2014b). Initial reports of the tooth phenotype simply mentioned that the relatives of some JEB patients had poor enamel, without recognition that the phenotype was truly AI (Murrell et al., 2007; Pasmooij et al., 2007). It was not until later that families segregating AI with autosomal dominant inheritance, and without any family members with JEB, were recognized and the phenotype more accurately described as AI (Poulter et al., 2014b).

The enamel of *LAMA3* and *LAMB3* patients is similarly described as hypoplastic, with grooving and pitting often present (Kim et al., 2013; Lee et al., 2014; Poulter et al., 2014b). Carriers of the JEB-causing *LAMA3* frameshift mutation c.488delG were found to have rough, pitted enamel due to haploinsufficiency of the protein (Yuen et al., 2012). One report of a patient carrying a *LAMB3* mutation highlighted that the multi-cusped teeth were more severely affected by AI than the anterior teeth (Kim et al., 2016b), although further study is required to determine whether this is a general trend.

AI-causing mutations in *LAMA3* and *LAMB3* present somewhat of a dichotomy. *LAMA3* variants that cause AI in heterozygous carriers, also cause JEB in biallelic individuals but the majority of AI-causing *LAMB3* variants have not been reported in JEB patients. Most *LAMB3* variants identified in AI patients are either frameshift or nonsense mutations predicted to escape NMD. The variants are consistent with a dominant gain of function disease mechanism, unlike the loss of function variants associated with recessively inherited JEB. However, Prasad et al. (2016a) did identify two AI patients carrying *LAMB3* mutations that do not fit this pattern of pathogenesis. These mutations have also been identified in JEB patients as recurrent mutations at hypermutable CpG sites (Kivirikko et al., 1996). Nevertheless, the pathogenicity of these variants in AI remains to be confirmed since segregation of one of the variants with the dental phenotype was inconsistent, and for the other, no segregation was possible, since only one affected individual was recruited to the study.

*COL17A1* mutation carriers with an AI phenotype harbor glycine substitutions, as well as nonsense, frameshift and splicing mutations (McGrath et al., 1996; Murrell et al., 2007). The nonsense and frameshift mutations identified would be expected to lead to NMD, suggesting that the cause of the phenotype is haploinsufficiency. The glycine substitutions are predicted to disrupt an extracellular collagenous triple helix, potentially affecting both the susceptibility of the protein to degradation and its secretion (McGrath et al., 1996).

No patients with AI and heterozygous *LAMC2* mutations have yet been reported although it seems likely that these exist. In addition, other genes which are known to be involved in the etiology of JEB, such as integrin, alpha 6 (MIM ^*^147556) (Ruzzi et al., 1997) and integrin, beta 4 (MIM ^*^147557) (Vidal et al., 1995), may harbor heterozygous mutations that cause dental defects in the absence of skin blistering since homozygous patients with a number of different JEB sub-types present with enamel hypoplasia (Fine et al., 2008).

#### AMTN

Amelotin (AMTN; MIM ^*^610912) is a proline, leucine, threonine and glutamine rich protein secreted by transition and maturation stage ameloblasts (Iwasaki et al., 2005; Moffatt et al., 2006). The protein localizes to the ameloblast basal lamina where it known to bind to itself, to ODAM (odontogenic, ameloblast associated) and to SCPPPQ1 (secretory calcium-binding phosphoproteins proline-glutamine rich 1) (Holcroft and Ganss, 2011; Fouillen et al., 2017). It is hypothesized to form large aggregates (Holcroft and Ganss, 2011; Bartlett and Simmer, 2015). These aggregates are thought to mediate attachment between the maturation stage ameloblasts and the mineralizing enamel (Moffatt et al., 2014). AMTN is also expressed at the junctional epithelium, a structure partially formed from maturation stage ameloblasts that mediates the attachment of the gingiva to the tooth (Bosshardt and Lang, 2005; Moffatt et al., 2006).

Murine models of amelotin function include an *Amel* promoter driven *Amtn* overexpressing mouse (p*Amel*:*Amtn*^+/+^) and a knockout (*Amtn*^−/−^) model (Lacruz et al., 2012a; Nakayama et al., 2015). The p*Amel*:*Amtn*^+/+^ model had thin brittle enamel with an irregular surface layer (Lacruz et al., 2012a). The *Amtn*^−/−^ model had mandibular incisors with a chalky appearance and enamel in which mineralization was delayed and organic material retained (Nakayama et al., 2015). The surface enamel easily chipped away and was found to be softer than the inner and middle enamel (Nakayama et al., 2015; Nunez et al., 2015). These phenotypes and an *in vitro* study that found that AMTN promotes HA precipitation, suggested a critical role for AMTN in the formation of compact surface aprismatic enamel during maturation (Abbarin et al., 2015). Therefore, AMTN could be bi-functional, with roles in both cell-matrix attachment and mineral nucleation.

In humans, only one *AMTN* mutation has been associated with AI; an in-frame deletion spanning exons 3 to 6 (Smith et al., 2016). The family exhibited hypomineralized AI with autosomal dominant inheritance. Phenotypic analysis of teeth revealed that the enamel was of a lower mineral density when compared to WT and the typical prismatic enamel structure was disturbed throughout the enamel layer.

#### FAM83H

Family with sequence similarity 83, member H (FAM83H; MIM ^*^611927), is an intracellular protein with ubiquitous expression (Lee et al., 2008a). In oral tissues, the ameloblasts show the highest expression of FAM83H, especially in pre-secretory and secretory stages (Lee et al., 2008a). Lower expression is seen in maturation stage ameloblasts as well as in the odontoblasts and alveolar bone (Lee et al., 2008a).

Through homology, FAM83H was suggested to be involved in membrane vesicle trafficking or cytoskeletal reorganization (Foster and Xu, 2003; Ding et al., 2009). FAM83H has now been shown, via binding to casein kinase 1 (CK1), to regulate the organization of the keratin cytoskeleton and therefore also to be involved in desmosome formation (Kuga et al., 2016).

Human mutations identified in *FAM83H* cause autosomal dominant hypocalcified AI (MIM #130900) (Mendoza et al., 2007; Kim et al., 2008). Analysis of teeth from individuals with *FAM83H* mutations identified defects in enamel rods, especially at the DEJ, with increased organic content within the enamel (El-Sayed et al., 2010; Zhang et al., 2015a).

All of the mutations identified so far have been located in the final, largest exon and all but two are nonsense or frameshift variants predicted to lead to premature translation termination (Kim et al., 2008; Lee et al., 2008a, 2011; Ding et al., 2009; Hart et al., 2009; Hyun et al., 2009; Wright et al., 2009, 2011; El-Sayed et al., 2010; Chan et al., 2011). As terminating mutations in the last exon are generally not subject to NMD (Nagy and Maquat, 1998), the truncated products may cause AI through a dominant gain of function effect.

*In vitro* analysis of three of the reported AI-causing *FAM83H* mutations suggests that the mutations alter the localization of the FAM83H protein, leading to an increased concentration within the nucleus rather than its typical cytoplasmic location (Lee et al., 2011). Analysis of the shortest of the truncated mutant proteins reported, p.S287^*^ (NP_198488.3), revealed that it bound and inhibited CK1 (Kuga et al., 2016). All of the twenty-seven *FAM83H* mutations identified reside within the first 1343 bp of the final exon (up to Glu694) with the final 1460 bp devoid of known mutations, suggesting that this region may not be critical to FAM83H function. In addition, some degree of phenotypic variation has been reported in patients, with mutations predicted to produce longer truncation products having a milder phenotype confined to just the cervical areas of the teeth (Wright et al., 2009).

Interestingly, *Fam83h* knockout mice and a mouse overexpressing FAM83H do not show an AI enamel phenotype (Kweon et al., 2013) supporting a dominant negative effect as the disease mechanism.

### Transport

The formation of enamel requires both the transport of large volumes of protein from ameloblasts via exocytic vesicles during the secretory stage and the removal of degraded protein via endocytic pathways during the maturation stage. During maturation, ameloblasts must also increase the active transport of mineral ions into the enamel space to support crystallite growth. Efficient and effective transport of cellular cargo is therefore critical in enamel formation.

#### WDR72

WD repeat domain 72 (WDR72; MIM ^*^613214) is believed to form a beta propeller structure (Jawad and Paoli, 2002; Valeyev et al., 2008) and is predicted by protein homology to be an intracellular vesicle coat protein (El-Sayed et al., 2009; Katsura et al., 2014). WDR72 expression is widespread, and is highest in bladder and kidney (Lee et al., 2010b). Immunolocalization of WDR72 in mouse incisors revealed more intense staining in maturation stage than secretory stage ameloblasts (El-Sayed et al., 2009), with a specific increase in expression noted to occur at the initiation of enamel maturation (Katsura et al., 2014).

*Wdr72* null mouse models exhibit hypomaturation AI (Katsura et al., 2014; Wang et al., 2015). In one model, maturation stage ameloblasts were shorter compared with WT mice, whereas there was no difference for secretory stage ameloblasts (Katsura et al., 2014). Affected enamel appeared stained and opaque and had retained proteins within it, including amelogenin. In another null mouse model, attachment between the ameloblasts and the enamel matrix was found to be disrupted (Wang et al., 2015). Ruffle ended maturation stage ameloblasts, thought to be responsible for protein removal, did not appear to be present and uptake of processed enamel matrix proteins by maturation stage ameloblasts was affected (Wang et al., 2015). In addition, the localization of putative Ca^2+^ transporter SLC24A4 was altered in *Wdr72*^−/−^ mice.

Originally, three truncating *WDR72* mutations were identified in patients with autosomal recessive hypomaturation AI (MIM #613211) (El-Sayed et al., 2009). Subsequently, other truncating mutations have been described, all causing an identical enamel phenotype and likely to be subject to NMD (Lee et al., 2010b; Chan et al., 2011; El-Sayed et al., 2011; Wright et al., 2011; Kuechler et al., 2012; Katsura et al., 2014). Patients with mutations affecting the region between the two beta propeller clusters have also been reported to exhibit hypodontia and delayed tooth eruption (Kuechler et al., 2012; Katsura et al., 2014). Additionally there have been reports of short stature in families with *WDR72* variants; however, given the consanguineous nature of the majority of the families studied and the lack of adequate controls, it is difficult to directly associate the phenotype with *WDR72* variants and to exclude the possibility that this is caused by an additional co-segregating variant.

#### SLC24A4

Solute carrier family 24 (Sodium/potassium/calcium exchanger), member 4 (SLC24A4; MIM ^*^609840) is one of a family of potassium dependent sodium/calcium exchangers. Members of this protein family share highly conserved hydrophobic regions, termed alpha-1 and alpha-2 repeats, which interact to form ion-binding pockets and lie within two clusters of five transmembrane helices (Iwamoto et al., 2000; Parry et al., 2013). SLC24A4 is highly expressed in a wide range of tissues including brain, aorta, lung and thymus (Li et al., 2002). Within the developing tooth, it is expressed by maturation stage ameloblasts and localizes to the membrane in contact with the developing enamel (Hu et al., 2012). In rat, *Slc24a4* transcripts are highly upregulated during the shift from secretion to maturation in the enamel organ (Lacruz et al., 2012b) and it is suggested that SLC24A4 is responsible for the active transport of Ca^2+^ ions from ameloblasts into the enamel matrix during maturation (Wang et al., 2014a). In mice, SLC24A4 expression is restricted to ruffle-ended maturation stage ameloblasts (Bronckers et al., 2015).

Mutations in *SLC24A4* cause a hypomaturation/hypomineralized AI phenotype with autosomal recessive inheritance (Parry et al., 2013; Seymen et al., 2014; Wang et al., 2014a; Herzog et al., 2015; Prasad et al., 2016a). Missense mutations affecting both alpha repeats and the cytoplasmic domain, as well as a nonsense mutation and a multi-exonic deletion, have been detected. Subsequent to the identification of *SLC24A4* mutations in human AI, incisors from *Slc24a4* null mice (Stephan et al., 2012) were examined using SEM and a similar phenotype was observed. The enamel was poorly mineralized and quickly wore away to expose the underlying dentine (Parry et al., 2013).

### Others: pH sensing, crystal nucleation, and unknown functions

Some of the genes known to be mutated in AI have protein products with functions that cannot be grouped easily. In other cases, their function is debated or is simply unknown. It is becoming ever more apparent that discovery of the causative mutations in AI is only the first step and that experiments to discover the functions of the encoded proteins through creation of murine models or *in vitro* study, will be crucial in the understanding of amelogenesis and the pathology of AI.

#### GPR68

G protein-coupled receptor 68 (GPR68; MIM ^*^601404) is a proton-sensing protein known to function in a wide range of cells including osteoblasts and osteocytes (Ludwig et al., 2003; Yang et al., 2006). Its activation leads to inositol phosphate formation and calcium release from intracellular stores (Ludwig et al., 2003). The protein contains seven transmembrane helices, with the histidine residues responsible for its pH sensing properties on the extracellular surface of the protein (Ludwig et al., 2003). GPR68 has been shown to be expressed in ameloblasts throughout all stages of amelogenesis, with strong expression at the ameloblast pole in contact with the enamel matrix (Parry et al., 2016). GPR68 activation is known to result in inositol phosphate formation that is associated with cytoplasmic re-organization and membrane ruffling (Honda et al., 1999; Czech, 2000; Parry et al., 2016). Therefore, Parry et al. (2016) proposed that GPR68 acts as a pH sensor in amelogenesis, directing ameloblasts to switch between the ruffle ended and smooth ended conformations during the maturation stage.

Three families with autosomal recessive hypomineralized AI have been reported with mutations in the single exon *GPR68* gene (MIM #617217) (Parry et al., 2016). All are predicted to result in loss of function. Two of the families carried deletions expected to remove histidine residues shown to be crucial to the pH sensitivity or the structural integrity of the protein. The third carried a missense variant predicted to destabilize the second transmembrane helix.

In light of mutations in *GPR68* leading to AI in humans, the incisors of *Gpr68*^−/−^ mice (Mogi et al., 2009) were assessed for enamel defects (Parry et al., 2016). A limited phenotype, of retarded formation of enamel with minor structural differences, was reported. This phenotype partially mirrors *GRP68*-associated AI in humans but may also reflect temporal differences in human and murine amelogenesis, the genetic strain of the *Gpr68*^−/−^ model or the possibility that mutant protein persists in cases of human AI.

#### C4orf26

The chromosome 4 open reading frame 26 (*C4orf26*; MIM ^*^614829) gene encodes a proline rich protein containing a signal peptide, two highly conserved but unknown motifs and ten predicted phosphorylation sites (Parry et al., 2012). It has been suggested, based on its amino acid sequence, that C4orf26 belongs to the acidic phosphoprotein family of proteins. These are known to promote HA crystallization, and C4orf26 peptide with a phosphorylated C-terminus was shown to promote HA nucleation and support crystal growth *in vitro* (Parry et al., 2012). Analysis of *C4orf26* expression in rat revealed transcripts in both secretory and maturation stage enamel organs but not in heart or kidney, and in a human cDNA panel lacking any dental tissues, expression was highest in placenta but was also evident in many other tissues (Parry et al., 2012).

Six mutations have been identified in *C4orf26* in ten families with autosomal recessive hypomineralized AI (MIM #614832) (Parry et al., 2012; Prasad et al., 2016b). These include only nonsense, frameshift and splice site variants predicted to escape NMD. However, the resulting proteins are predicted to be non-functional. Enamel from affected individuals contained thin crystallites more typical of the early maturation stage of enamel development rather than of mature enamel. To date no mouse model has been characterized.

#### ACPT

Acid phosphatase, testicular (ACPT; MIM ^*^606362) is one of a group of enzymes capable of hydrolysing esters of orthophosphoric acid in acidic conditions (Romas et al., 1979). It is expressed by secretory, but not maturation stage, ameloblasts (Seymen et al., 2016) and it has been suggested to elicit odontoblast differentiation and mineralization by supplying phosphate during dentine formation (Choi et al., 2016). *ACPT* was first identified as a gene immediately centromeric to the kallikrein gene cluster within a region of chromosome 19q13.3-q13.4 that is differentially expressed in solid tumors (Yousef et al., 2001). *ACPT* is itself known to show lower expression in testicular cancer tissues compared to normal tissues and is regulated by steroid hormones (Yousef et al., 2001). Its chromosomal location, around 111 kb proximal to *KLK4*, is notable, although *Acpt* was first associated with amelogenesis through the differential analysis of ameloblast gene expression in rats (Lacruz et al., 2011). However, the function of ACPT during amelogenesis is currently unclear and no mouse model has been characterized to date.

ACPT is also expressed in the brain, where it is enriched at post-synaptic sites, and is thought to dephosphorylate the ErbB4 receptor (Fleisig et al., 2004). The ErbB4 receptor has important roles in neuronal differentiation and synaptogenesis, and ACPT acts as a tyrosine phosphatase to modulate signals mediated by ErbB4 that are important for neuronal development and synaptic plasticity (Fleisig et al., 2004).

Mutations in *ACPT* were recently identified in eight families with autosomal recessive hypoplastic AI (MIM #617297) (Seymen et al., 2016; Smith et al., 2017b). All seven variants identified are missense changes predicted to affect residues within the extracellular domain that makes up the majority (residues 29–390; NP_149059.1) of the 426 amino acid protein. Assessment of an affected tooth by X-ray computed tomography revealed that the enamel, where present, was around one tenth of the thickness of control enamel, but was well mineralized (Smith et al., 2017b). The authors found that the underlying dentine was hypermineralized compared to control, although no abnormalities were evident upon clinical examination (Smith et al., 2017b). Assessment of additional samples will be required to confirm this.

### Master controllers of amelogenesis

These genes control gene expression or the modification of a subset of AI- and other- proteins to influence enamel development. Both members of this group are involved in the development and/or maintenance of other organs and therefore disease can present as both isolated and syndromic AI, although enamel symptoms are usually the first to develop and can serve as an early warning for the development of other pathology.

#### FAM20A

Family with sequence similarity 20, member A (FAM20A) is one of a family of three human homologs of the *Drosophila* four jointed (fj) protein kinase. Additional family member FAM20C is a Golgi casein kinase responsible for phosphorylating many secreted proteins involved in biomineralization (Tagliabracci et al., 2012). *In vitro* expression of FAM20A has shown that it is also located in the Golgi (Ishikawa et al., 2012; Wang et al., 2013a). FAM20A has been shown to control FAM20C localization and to potentiate its extracellular action *in vitro* (Ohyama et al., 2016). The protein is therefore designated a pseudokinase (Cui et al., 2015).

Mutations in *FAM20A* have been shown to cause autosomal recessive AI and gingival fibromatosis syndrome (AIGFS; O'Sullivan et al., 2011) and enamel renal syndrome (ERS; Jaureguiberry et al., 2012; Wang et al., 2013a). Both syndromes have since been recognized as phenotypic variants of the same condition and are now collectively termed ERS (MIM #204690) (de la Dure-Molla et al., 2014). Patients with *FAM20A* mutations have hypoplastic AI which, in extreme cases, may present as a complete absence of enamel (de la Dure-Molla et al., 2014) or characteristic “glassy” incisor teeth. There are a variety of associated ERS oral defects that may include delayed tooth eruption, hyperplastic dental follicles, pulp stones and gingival overgrowth with ectopic calcification (de la Dure-Molla et al., 2014). Calcification of other organs, most frequently nephrocalcinosis, is variably reported. This may be due to a combination of age, genetic modifiers, and exposure to Ca^2+^ channel blocking drugs (Poulter et al., 2015).

The symptoms associated with ERS suggest that FAM20A phosphorylates proteins involved in enamel formation and other aspects of tooth development, as well as those critical to Ca^2+^ regulation within the kidney. Analysis of developing murine oral tissues showed the presence of FAM20A within ameloblasts, odontoblasts and at consistently high levels within the oral epithelium (Wang et al., 2014c). Ameloblasts expressed FAM20A only during the secretory stage, consistent with the hypoplastic AI phenotype (Wang et al., 2014c). Analysis of teeth from *Fam20a*^−/−^ mice showed that the ameloblast layer was disorganized and detached from the DEJ, leading to pitted and thin enamel (Vogel et al., 2012). A similar phenotype was observed in an epithelial specific (K14-cre) *Fam20a*^−/−^ model, along with reduced levels of ENAM and MMP20 proteins in ameloblasts but increased levels of AMBN (Li et al., 2016). The authors suggest that reduced levels of ENAM may be due to a lack of phosphorylation by FAM20C due to the absence of FAM20A. However, the authors do not explain the increased and decreased levels of AMBN and MMP20, respectively.

#### DLX3

Distal-less homeobox 3 (DLX3; MIM ^*^600525) is one of a family of six DLX transcription factors essential to the development of placental, epidermal and ectodermal appendages (Beanan and Sargent, 2000). DLX3 is also expressed in skin, bone and dental tissues including both the odontoblasts and ameloblasts, with greatest expression in ameloblasts during the late secretory stage (Zhang et al., 2015b). Murine whisker and hair follicles strongly express DLX3 before birth but expression decreases after birth. DLX3 is expressed in the placenta during early embryonic development and *Dlx3*^−/−^ mice die around embryonic day 10 due to placental defects, whereas *Dlx3*^+/−^ mice appear phenotypically normal (Morasso et al., 1999). Although, the role of DLX3 has been studied in dentine, bone and hair, through the creation of conditional knockouts of *Dlx3*, (Hwang et al., 2008; Duverger et al., 2012, 2013) no study has reported the creation of a conditional knockout of *Dlx3* in ameloblasts.

Mutations in *DLX3* are known to cause the autosomal dominantly-inherited trichodentoosseous syndrome (TDO; MIM #190320) and amelogenesis imperfecta hypomaturation-hypoplastic type with taurodontism (AIHHT; MIM #104510). TDO is defined as AIHHT with additional features such as kinky hair, especially in infancy, and increased bone density/thickening of the craniofacial cortical bones (Lichtenstein and Warson, 1971). Other variably associated features include flattened fingernails and altered craniofacial morphology, including dolichocephaly and prognathism (Price et al., 1998).

A mutation was first associated with AIHHT in patients carrying a 2 bp deletion (c.561_562delCT; NM_005220.2) in *DLX3* (Dong et al., 2005). Other families of different ethnicities have since been described with the same *DLX3* mutation, potentially highlighting the position as a mutational hotspot. However, the phenotype varies from AI with only mild taurodontism (Kim et al., 2016a) to TDO (Lee et al., 2008b) suggesting that there may be wide phenotypic variation in presentation, even for identical *DLX3* mutations.

DLX3, like all DLX proteins, contains a homeobox domain flanked by N- and C-terminal transactivation domains. The homeobox domain directly binds to target DNA sequences but the transactivation domains have also been shown to be crucial to this binding. DLX3 has been shown to bind to the enhancer regions of *Amelx, Enam* and *Odam* in a murine ameloblast cell lineage and to positively regulate their expression (Zhang et al., 2015b).

Only six *DLX3* variants have been reported so far, including two frameshift variants predicted to escape NMD but to result in an altered C-terminal transactivation domain and four missense variants predicted to affect residues (spanning residues 133–182; NP_005211.1) within the central DNA binding homeodomain (residues 129–188). Disease has been postulated to result from haploinsufficiency (Nieminen et al., 2011) but other effects, including dominant negative through binding to WT DLX3 (Duverger et al., 2008), as well as gain of function, through interactions of the mutant protein with other transcription factors, have not been ruled out.

### Frequency of mutations identified in AI cohorts

Given the paucity of epidemiological data on AI, the difficulty in estimating its prevalence and the recent advances in knowledge of the underlying genetic causes, we assessed the frequencies of variants in each gene recorded in the AI Leiden Open Variant Database (LOVD) resource (http://dna2.leeds.ac.uk/LOVD/). These findings are biased by studies focusing on particular populations or the specific recruitment of consanguineous families with recessively inherited AI for ease of study. The targeted sequencing of AI cohorts for variants in particular genes and the time since discovery of causality of each of the AI genes will also influence reported relative contributions to disease burden. It is also likely that variants that cause AI as a dominant trait but JEB as a recessive trait, i.e. those in *LAMA3, COL17A1*, and potentially also *LAMB3*, are underreported. JEB carriers are often only mentioned in passing in reports as having poor enamel. A larger AI-focused study of carriers of *LAMA3, LAMB3*, and *COL17A1* variants would help to clarify the frequency of enamel defects in these individuals and whether the same variants cause both isolated AI and JEB in the case of *LAMB3* variants.

However, with all of these caveats, as of 23rd May 2017, the AI LOVD resource (http://dna2.leeds.ac.uk/LOVD/) details 192 different, published AI gene variants identified in 270 families with AI (Table 1). Analysis of these variants shows that just four genes account for the cause of AI in 163 families (60.4%). Variants were most commonly identified in *FAM83H* (19.3% of cases), followed by *FAM20A* (15.2%), *ENAM* (14.2%), and *AMELX* (11.5%). Of the AI families reported with a known mutation, 132 (48.9%) have autosomal dominant AI, with autosomal recessive (109 families; 40.4%) and X linked inheritance (31 families; 11.5%) less frequently reported.

**Table 1:** Summary of the variants reported in individuals with AI and entered in the AI LOVD.

**Gene (Refseq refs)**	**No. of variants**	**Families no. (%)**	**Variants identified in ≥3 families; number of families: reported nationality/ethnicity**	**Mode of inheritance and trends in variants identified**
*FAM83H* NM_198488.3 NP_940890.3	26	52 (19.3)	c.973C>T, p.(L31R), 3: Korean and Chinese; c.1192C>T, 5: Korean, Turkish, Caucasian, N/R; c.1354C>T, p.(Q452^*^), 5: Korean, Danish, Chinese, Jewish, Caucasian; c.2029C>T, p.(Q677^*^), 5: Korean, Caucasian, N/R.	AD. All within final exon. All except c.1669G>T, p.(G557C), are frameshift/nonsense variants predicted to escape NMD.
*FAM20A* NM_017565.3 NP_060035.2	42	41 (15.2)	c.34_35delCT, p.(L12Afs^*^67), 5: Pakistani, Moroccan, Korean/Turkish, N/R; c.406C>T, p.(R136^*^), 3: Brazilian, Omani, Iranian.	AR. Most are nonsense/frameshift. Three missenses reported: c.518T>G, p.(L173R), c.992T>G, p.(G331D), c.1207G>A, p.(D403N).
*ENAM* NM_031889.2 NP_114095.2	18	39 (14.4)	c.92T>G, p.(L31R), 5 families: British; c.157A>T, p.(K53^*^), 7: Swedish; c.588+1delG, p.?, 5: Japanese, Lebanese, Slovenian, N/R; c.1259_1260insAG, p.(V422Pfs^*^27), 7: Turkish, Slovenian, N/R.	AD (AR also reported). Most are protein-truncating. In-frame indels and missense variants also identified.
*AMELX* NM_182680.1 NP_872621.1	22	31 (11.5)	c.208C>A, p.(P70T), 6: North American, N/R; c.473delC, p.(P158Hfs^*^31), 3: North American, British.	XLD. Most are protein-truncating variants. Missense variants affect the N-terminus.
*DLX3* NM_005220.2 NP_005211.1	6	15 (5.6)	c.561_562delCT, p.(Y166Qfs^*^13), 4: Australian, Korean, Caucasian, Turkish; c.571_574delGGGG, p.(G191Rfs^*^66), 7: North American.	AD. Most are missenses in homeodomain, other two are frameshifts predicted to escape NMD and to affect C-terminal transactivation domain.
*MMP20* NM_004771.3 NP_004762.2	11	13 (4.8)	c.389C>T, p.T130I, 3: French, N/R c.678T>A, p.H226Q, 3: Turkish, North American, N/R. c.954-2A>T, p.?, 4: North American, French/German/Moroccan.	AR. Variety of missense, frameshift, splice and nonsense variants reported.
*WDR72* NM_182758.3 NP_877435.3	10	12 (4.4)	c.1467_1468delAT, p.(V491Dfs^*^8), 3: Mexican, Turkish, unknown.	AR. All are nonsense/frameshift predicted to under-go NMD, except missense variant c.182A>G, p.(H61R).
*COL17A1* NM_000494.3 NP_000485.3	10	12^*^^∧^ (4.4)	N/A	AD. Nonsense/frameshift variants predicted to undergo NMD, and missense variants affecting glycine residues.
*C4orf26* NM_178497.3 NP_848592.2	6	10^*^ (3.7)	c.51_56delGGTAACinsATGCTGGTTACTGGTA, p.(V18Cfs^*^23), 3: North American; c.229C>T, p.(R77^*^), 4: Omani.	AR. All nonsense/frameshift/splice variants. Nonsense/frameshifts predicted to escape NMD.
*LAMB3* NM_000228.2 NP_000219.2	9	9 (3.3)	N/A	AD. Majority frameshift/nonsense. Most lead to stop codon in penultimate/final exon. Two variants within other exons identified.
*KLK4* NM_004917.4 NP_004908.4	4	9 (3.3)	c.632delT, p.(L211Rfs^*^37), 5: Pakistani.	AR. All are nonsense/frameshift. Not all are predicted to undergo NMD.
*ACPT* NM_033068.2 NP_149059.1	7	8 (3.0)	c.713C>T, p.(S238L), 3: Turkish.	AR. All missense variants within the extracellular domain.
*SLC24A4* NM_153646.3 NP_705932.2	5	6 (2.2)	N/A	AR. Variety reported, including a multi-exon deletion, a nonsense and missense variants.
*ITGB6* NM_000888.*4* NP_000879.2	6	5 (1.9)	N/A	AR. All missense variants except one nonsense variant, c.1846C>T, p.(R616^*^).
*LAMA3* NM_000227.3 NP_00218.2	4	4^∧^ (1.5)	N/A	AD. All frameshift, nonsense or splice variants.
*GPR68* NM_001177676.1 NP_001171147.1	3	3 (1.1)	N/A	AR. One frameshift, one in-frame deletion, one missense variant.
*AMBN* NM_016519.5 NP_057603.1	2	2 (0.7)	N/A	AR. One in-frame exon deletion, one splice variant.
*AMTN* NM_212557.3 NP_997722.1	1	1 (0.4)	N/A	AD. One variant: a multi-exon, in-frame deletion.
Total	192	270^∧^		

*Genes are ordered in decreasing number of families reported. No. of variants denotes the number of unique variants reported for each gene within the AI LOVD. Nationality/ethnicity is as reported, in some cases, multiple families are reported within one study with only details of the nationalities/ethnicities of the cohort included but with no specific nationality/ethnicity assigned to each individual/family. In such cases, all nationalities/ethnicities have been listed. The inheritance of disease due to ENAM variants has been reported to be AR in some instances, however heterozygous and biallelic individuals for ENAM variants were reported to have a milder and more severe phenotype, respectively*.

* *one family carries variants in both COL17A1 and C4orf26*.

∧ *one family carries variants in both COL17A1 and LAMA3. Therefore, total number of families is 270 not 272. AD, autosomal dominant; AR, autosomal recessive; NMD, nonsense mediated decay; N/A, not applicable; N/R, not reported; XLD, X-linked dominant. Data obtained from AI LOVD: http://dna2.leeds.ac.uk/LOVD/ 23rd May 2017*.

Analysis has also shown that there are a number of variants that have been repeatedly identified in families with AI and that may represent founder mutations or reside at mutational hotspots. These include *ENAM* c.92T>G, p.(L31R) (Brookes et al., 2017) and *KLK4* c.632delT, p.(L211Rfs^*^37) (Smith et al., 2017a). Some genes, for example *FAM83H* and *WDR72*, show clear trends in the types and positions of AI-causing variants reported, giving clues as to how these variants cause disease.

From a recent focused clinical exome study, molecular diagnoses were obtained in only 18 of 65 syndromic and non-syndromic AI cases (27.7%) (Prasad et al., 2016a). Earlier studies using Sanger sequencing of candidate genes identified variants in 36.6–48.7% of families (Chan et al., 2011; Wright et al., 2011). This suggests that additional AI genes remain to be identified and/or that variants in known AI genes, such as intronic, regulatory, and larger structural alterations, are being missed by current analysis pipelines. For example, particular variant types, such as heterozygous copy number changes larger than an exon, are likely to be under-represented in reports since they would not have been identified by Sanger sequencing or WES data without specific downstream analysis (Poulter et al., 2015; Smith et al., 2016).

## Discussion

This article reviews the genes and proteins where variants cause AI presenting in isolation of other health problems and also reviews current knowledge of their functions. Potential mechanisms of disease were explored with reference to evidence from mouse models and human pathology. Finally, the prevalence of reports of families with AI with mutations in each gene was explored through the development and interrogation of an AI LOVD resource.

It is evident that some proteins with roles in amelogenesis can be classified into clear functional groups, with obvious examples including the EMPs: AMELX, ENAM and AMBN; the enamel matrix proteases: MMP20 and KLK4; as well as those involved in cell-cell and cell-matrix adhesion: ITGB6, LAMB3, LAMA3, COL17A1, AMTN, and FAM83H, transport: WDR72 and SLC24A4 and master controllers of amelogenesis: FAM20A and DLX3. However, there is also an emerging group of proteins that exhibit diverse functions in enamel development. These include proteins thought to be involved in activities as wide-ranging as crystal nucleation (C4orf26), proton sensing (GPR68) and those with unknown roles (ACPT). Further investigation is required to define the role of each protein in amelogenesis and to identify interacting partners, mechanisms, and functional pathways.

**Figure 4:**
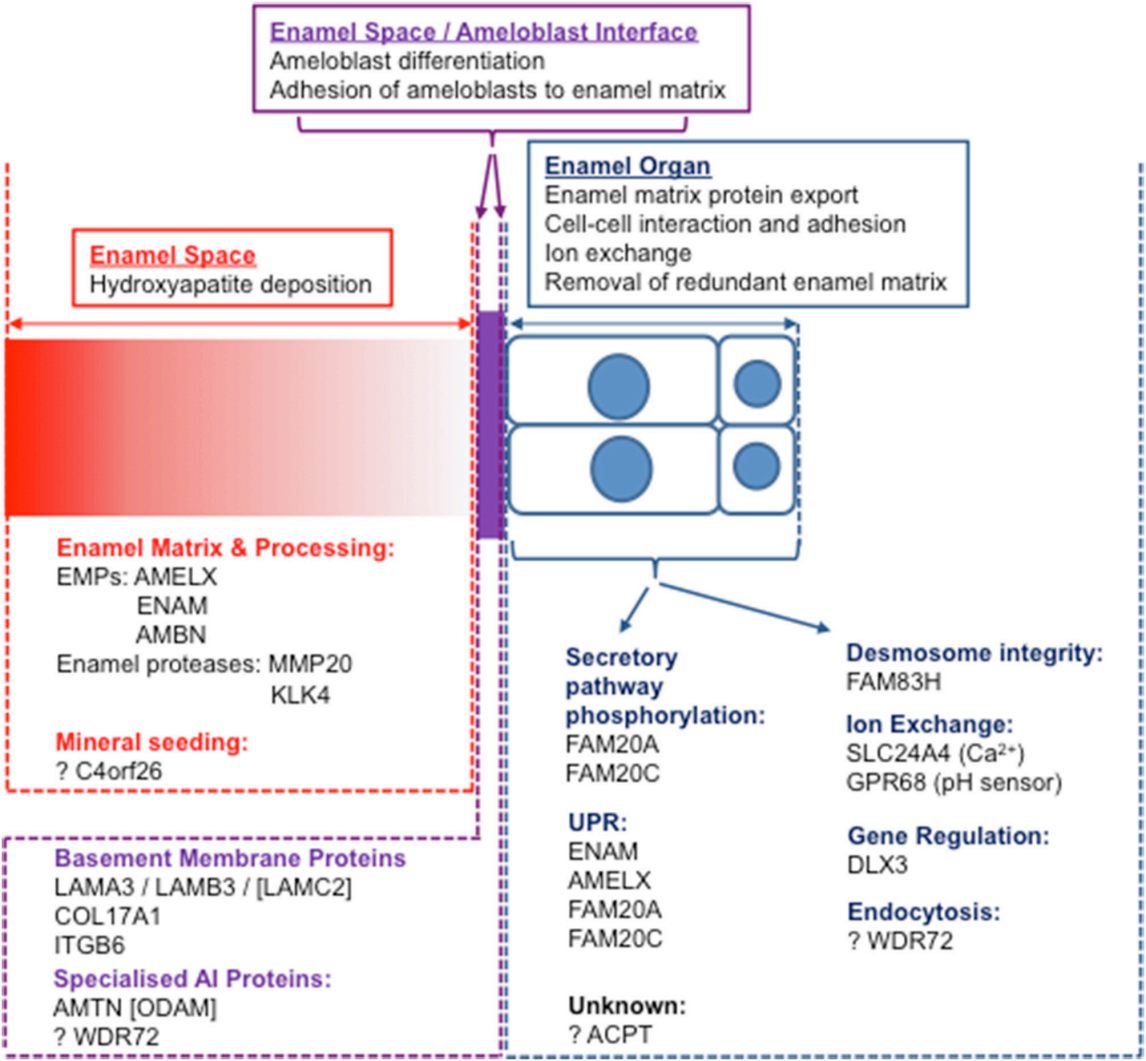
The three “compartments” involved in amelogenesis: the enamel space (extracellular matrix; red), the enamel organ (ameloblast cell layer and supporting cells; blue) and the interface between the two (purple). Known AI proteins are grouped according to functions within compartments. Those with no causative mutations for AI identified are designated by square brackets.

To understand the events of amelogenesis and how mutations can impact on the enamel formed, it is important to consider three linked but distinct compartments, namely the cellular enamel organ, including both the ameloblast cell layer and its supporting cells, the extracellular enamel space and the interface between sites (Figure 4). Initial mutation discovery focused on EMPs and the events in the extracellular enamel space. However, it is becoming clear that intracellular events in the ameloblast, ameloblast cell-cell interactions and ameloblast attachment to the enamel matrix also play critical roles in amelogenesis. Even disease previously considered to be entirely the result of perturbations in extracellular events, such as that resulting from EMP mutations, has, in some cases, been shown to result in catastrophic intracellular effects. For example, some mutations in *Amelx* (Barron et al., 2010) and *Enam* (Brookes et al., 2017) lead to activation of the unfolded protein response and to apoptosis of ameloblasts. Therefore, it is important to consider pathology in the context of all three compartments and to study the roles of the affected proteins through the production of murine models with AI specific mutations. Assessment of the variants identified in human AI to date suggests that for many AI genes, trends indicate that protein absence may not be the mechanism of disease.

Classification of AI by phenotype and pattern of Mendelian inheritance has been modified since the first description of AI as a separate condition to dentinogenesis imperfecta in 1938 (Finn, 1938; Aldred et al., 2003). In recent years, the narrow definition of AI as an isolated enamel pathology has expanded to include diverse syndromes with generalized developmental enamel defects indistinguishable from AI in isolation (Aldred et al., 2003). The ability to identify the underlying genetic cause in individuals with AI has informed a more meaningful classification. Nevertheless, recognition of characteristic phenotypes remains useful. For example, patients with *FAM20A* variants can be readily identified upon oral examination. Patients with *FAM20A* variants should be referred for specialist renal evaluation and follow-up, to better understand the natural history of this feature and for development of intervention strategies to limit development of ectopic calcification. The contribution of this gene to the variant load reported for AI, described in the LOVD resource, is 15.2%. Therefore, *FAM20A* variants are the second most commonly reported cause of AI overall and the most commonly reported cause for autosomal recessive AI, at least in the AI LOVD of published reports. This finding highlights the real need for genetic diagnosis for AI patients. Knowledge of AI genetics has already prompted the development of a targeted diagnostic AI genetic screen within the UK National Healthcare Service that will help to inform improved patient pathways and to raise standards of care (Holland, 2017).

WES has expedited the identification of new genetic variants that cause AI (O'Sullivan et al., 2011; Jaureguiberry et al., 2012; Poulter et al., 2014a,b) but it is likely that more genes, not currently known to be critical for enamel formation, remain to be identified. As costs continue to fall, whole genome sequencing is also facilitating the discovery of mutations that cannot be found by conventional WES analysis (Poulter et al., 2015). For many human conditions, greater awareness of the importance of non-coding mutations is leading to an increase in the study of patient mRNA. However, for AI this approach is hampered by the enamel specific expression of many of the genes so far implicated. Transcript profiling in mammalian models can overcome this difficulty and highlight potential candidate genes, such as *SLC24A4*, where genetic variants were subsequently confirmed as causing of AI (Lacruz et al., 2012b).

The identification of AI genes will allow clinical and molecular diagnoses to be matched. This will in turn help clinicians to improve patient pathways and offer a more accurate prognosis and clearer risk information to patients and other family members. Genotyping of AI cohorts will facilitate participant recruitment to future clinical trials to develop improved clinical decision-making for AI, consistent with an increasingly personalized precision approach to care. To further support the development of diagnostic screening and variant interpretation in AI, we have created a dedicated LOVD resource recording all published mutations causing AI presenting in the absence of other health problems (http://dna2.leeds.ac.uk/LOVD/). The improved understanding of AI resulting from identification of the causative mutations and their pathogenesis also highlights new avenues for possible therapeutic intervention to improve outcomes for those with AI. ER stress is now recognized as a mechanism of AI pathogenesis (Brookes et al., 2014, 2017) and represents a possible therapeutic opportunity through *in utero*/early post-natal use of protein chaperoning/anti-apoptotic drugs such as 4-phenylbutyrate (Brookes et al., 2014). Biomimetic technologies that repair carious lesions to enamel have also been reported (Brunton et al., 2013) although it remains to be seen whether these will be effective in treating selective forms of AI. A thorough understanding of the molecular mechanisms underlying biomineralization, obtained through a genetic dissection of AI, will inform the development of new interventions for enamel defects.

In summary, recent increases in our understanding of the genetic variants that cause AI offer us new insights into the varied cellular and extracellular biological processes that are essential for enamel formation. Collation of published genetic variants causing AI presenting in the absence of other health problems in one LOVD resource will support this process. New genetic insight can translate into improved patient care in the short-term and will inform the development of new therapeutic strategies for enamel pathologies that are not expected to be restricted to AI in the future.

## Author contributions

CELS drafted the manuscript, figures and tables, deposited variants in the AI LOVD and curates the AI LOVD. AA hosts the AI LOVD. JAP, CFI and AJM revised early drafts of the manuscript. JK, SJB and AJM drafted figures. All authors read, critically revised and gave approval for the manuscript. All authors agree to be accountable for all aspects of the work.

### Conflict of interest statement

The authors declare that the research was conducted in the absence of any commercial or financial relationships that could be construed as a potential conflict of interest.
