# Supplementary material for: Amelogenesis Imperfecta; Genes, Proteins, and Pathways
Source: Front Physiol. 2017 Jun 26;8:435. doi: 10.3389/fphys.2017.00435 (PMC5483479; doi:10.3389/fphys.2017.00435)
Supplement: Supplementary file 1 [file Table1.docx]

Supplementary Material

**Amelogenesis Imperfecta; Genes, Proteins And Pathways**

**Claire E. L. Smith^*^, James A. Poulter, Agne Antanaviciute, Jennifer Kirkham, Steven J. Brookes, Chris F. Inglehearn and Alan J. Mighell**

*** Correspondence:** Claire Smith: c.e.l.smith@leeds.ac.uk

| **Gene** | **Proposed function in amelogenesis** | **Mouse enamel phenotype (selected models)** | **Mouse model references** |
| --- | --- | --- | --- |
| Amelogenin, X linked (*AMELX*) | EMP  Structural component of developing enamel matrix.  Alternative splicing and cleavage produces peptides with distinct roles. | ***Amelx^Y/-^***  <10% of WT enamel volume.  Enamel lacked normal prismatic structure (“Flat, plate like”).  ***Amelx^Y/Y64H^* (*M100888*)**  Roughened, opaque, chalky white enamel. | [Gibson et al. (2001](#_ENREF_6))  [Masuya et al. (2005](#_ENREF_14))  [Barron et al. (2010](#_ENREF_2)) |
| Ameloblastin (*AMBN*) | EMP  Influences ameloblast proliferation and differentiation.  Undergoes PTM and cleavage to form products with distinct roles, including Ca^2+^ binding.  Possible roles in cell adhesion and extracellular signalling. | ***Ambn^-5,6/-5,6^***  Severely hypoplastic enamel.  Ameloblasts lost contact with the enamel matrix.  Tomes’ processes failed to develop.  ***p.Krt14:Ambn^+/+^***  Altered rod structure.  Loss of surface aprismatic enamel.  Altered craniofacial development. | [Paine et al. (2003](#_ENREF_20))  [Fukumoto et al. (2004](#_ENREF_5))  [Smith et al. (2009](#_ENREF_27))  [Wazen et al. (2009](#_ENREF_32))  [Atsawasuwan et al. (2013](#_ENREF_1)) |
| Enamelin (*ENAM*) | EMP  Undergoes cleavage by proteinases to form peptides with distinct roles, including binding HA crystals. | ***Enam* LoF**  Hypomaturation AI in heterozygotes.  Total lack of enamel in homozygotes.  ***Enam^-1-7/-1-7^***  Homozygotes lacked true enamel.  Heterozygotes had discoloured incisors that wore rapidly.  ***Enam*^p.S55I/p.S55I^**  Homozygotes had smooth white enamel surface, Tomes’ process absent, ovoid, irregular cell masses, cells vacuolated and filled with vesicular structures.  Heterozygotes had patches of white, opaque, chalky tissue, disorganized Tomes’ process, ameloblasts lost contact with enamel matrix, formed cyst-like structures. | [Masuya et al. (2005](#_ENREF_14))  [Seedorf et al. (2007](#_ENREF_24))  [Hu et al. (2008](#_ENREF_7))  [Brookes et al. (2017](#_ENREF_3)) |
| Matrix metalloproteinase 20 (*MMP20*) | Secretory stage cleavage of EMPs.  Activation of KLK4 by cleavage.  Mediation of ameloblast cell movement by cleavage of extracellular domains of cadherins.  May affect ameloblast development. | ***Mmp20^-/-^***  Defective AMELX processing.  Loss of the typical rod structure.  Reduced mineral content.  Enamel softer than WT.  ***pAmelx:Mmp20*^+/+^**  Hypomineralised enamel.  Large amounts of smaller EMP cleavage products. | [Caterina et al. (2002](#_ENREF_4))  [Shin et al. (2014](#_ENREF_25))  [Hu et al. (2016](#_ENREF_8)) |
| Kallikrein-related peptidase 4 (*KLK4*) | Maturation stage enamel matrix proteinase.  Further degrades peptide cleavage products produced by MMP20.  Inactivation of secretory stage proteinase, MMP20. | ***Klk4^-/-^***  Hypomaturation AI.  Enamel crystallites did not function as a unit.  Enamel easily abraded and breaks just above DEJ. | [Simmer et al. (2009](#_ENREF_26))  [Yamakoshi et al. (2011](#_ENREF_33))  [Hu et al. (2016](#_ENREF_8))  [Núñez et al. (2016](#_ENREF_19)) |
| Integrin, beta 6 (*ITGB6*) | Cell surface adhesion receptor.  Mediates cell-cell and cell-extracellular matrix interactions.  Able to activate TGF-β1 and other signalling molecules.  Localises to maturation stage ameloblasts. | ***Itgb6^-/-^***  Hypomineralised AI with disorganised enamel prisms and pits.  Retained AMELX within the enamel. | [Mohazab et al. (2013](#_ENREF_16)) |
| Laminin, alpha 3 (*LAMA3*) | Control of ameloblast differentiation and adhesion to the enamel surface. | ***Lama3^-/-^***  Smaller ameloblasts.  Abnormal enamel deposition.  Disorganised reduced enamel epithelium. | [Ryan et al. (1999](#_ENREF_23)) |
| Laminin, beta 3 (*LAMB3*) | Control of ameloblast differentiation and adhesion to the enamel surface. | ***Lamb3^-/-^***  Homozygotes die within 24hr of birth.  No assessment of the teeth carried out. | [Kuster et al. (1997](#_ENREF_11)) |
| Collagen, type XVII, alpha-1 (*COL17A1*) | Ligand of LM-332 (heterotrimeric protein consisting of LAMA3, LAMB3 and LAMC2).  See *LAMA3* / *LAMB3.* | ***Col17a1^-/-^***  No tooth phenotype described. | [Hurskainen et al. (2012](#_ENREF_9)) |
| Amelotin (*AMTN*) | Formation of compact aprismatic enamel.  May mediate attachment between ameloblasts and enamel. | ***Amtn^-/-^***  Chalky maxillary incisors.  Rough, irregular surface enamel that is easily chipped away.  ***pAmelx:Amtn*^+^*^/+^***  Brittle, thin enamel.  Defective enamel surface layer. | [Lacruz et al. (2012](#_ENREF_13))  [Nakayama et al. (2015](#_ENREF_18))  [Núñez et al. (2016](#_ENREF_19)) |
| Family with sequence similarity 83, member H (*FAM83H*) | Associates with perinuclear vesicles.  Cytoskeletal reorganisation, desmosome formation. | ***Fam83h^-/-^***  Majority die within 2 weeks.  No defects in enamel or dentine.  Hair present around base of teeth, scruffy coat.  Smaller pulp volume.  ***pActb:Fam83h^+/+^***  No defects in enamel or dentine. | [Kweon et al. (2013](#_ENREF_12))  [Wang et al. (2016](#_ENREF_31)) |
| WD repeat-containing protein 72 (*WDR72*) | Endocytosis by maturation stage ameloblasts.  Removes EMP degradation products to allow enamel to mineralise. | ***Wdr72^-/-^***  Hypomaturation AI.  EMP degradation products retained within the enamel during maturation.  RE may not form.  Enamel remains unmineralised.  Disrupted cell matrix attachment to the enamel matrix during maturation. | [Katsura et al. (2014](#_ENREF_10))  [Wang et al. (2015](#_ENREF_30)) |
| Solute carrier family 24 (sodium/  potassium/  calcium exchanger), member 4 (*SLC24A4*) | Transport of Ca^2+^ by RE. | ***Nckx4^-/-^***  Enamel breaks off incisor after eruption to expose underlying dentine. | [Stephan et al. (2012](#_ENREF_28))  [Parry et al. (2013](#_ENREF_21)) |
| G-protein coupled receptor 68 (*GPR68*) | Functions as a pH sensor.  Expressed throughout amelogenesis. | ***Ogr1^-/-^***  No major enamel defects.  Incisor enamel structure mildly affected.  Delayed yellowing of maxillary incisor teeth. | [Mogi et al. (2009](#_ENREF_15))  [Parry et al. (2016](#_ENREF_22)) |
| Chromosome 4 open reading frame 26 (*C4orf26*) | Unknown.  Promotes nucleation and crystallisation *in vitro.* | No model yet characterised. | N/A |
| Acid phosphatase, testicular (*ACPT*) | Unknown  Secretory stage expression  May supply phosphate during mineralisation. | No model yet characterised. | N/A |
| Family with sequence similarity 20, member A (*FAM20A*) | Controls localisation of Golgi casein kinase FAM20C and may potentiate its action.  Pseudokinase | ***Fam20a^-/-^***  Ameloblast layer disorganised and detached from the DEJ.  Pitted, thin enamel. | [Vogel et al. (2012](#_ENREF_29)) |
| Distal-less homeobox 3 (*DLX3*) | Transcription factor known to bind to the enhancers of *Amelx*, *Enam* and *Odam* and to positively regulate their expression. | ***Dlx3^-/-^***  Homozygotes die at E9.5-10  Heterozygotes do not exhibit an enamel phenotype. | [Morasso et al. (1999](#_ENREF_17)) |

Supplementary Table 1: AI gene function and relevant mouse models (if described). The proposed roles of the proteins encoded by genes in which mutations are reported to cause non-syndromic AI and their respective mouse models and phenotypes are described. Note that for some AI genes, no mouse model has been described to date. Mouse models are defined using standard nomenclature where possible. Overexpression models, where the promoter of one gene is driving the expression of another, are denoted: p(gene promoter driving expression):(gene under control). Where specific exons have been removed this is reflected in the nomenclature used. For *Amelx*, which is located on the X chromosome, the nomenclature used refers to hemizygous male mutants. Abbreviations used: DEJ dentine enamel junction; E embryonic day; EMP enamel matrix protein; HA hydroxy(l)apatite; hr hour; LOF loss of function; N/A not applicable; PTM post translational modification; RE ruffle ended ameloblasts; WT wild-type.

**References**

Atsawasuwan, P., Lu, X., Ito, Y., Zhang, Y., Evans, C.A., and Luan, X. (2013). Ameloblastin inhibits cranial suture closure by modulating MSX2 expression and proliferation. *PLoS One* 8(4)**,** e52800. doi: 10.1371/journal.pone.0052800.

Barron, M.J., Brookes, S.J., Kirkham, J., Shore, R.C., Hunt, C., Mironov, A., et al. (2010). A mutation in the mouse Amelx tri-tyrosyl domain results in impaired secretion of amelogenin and phenocopies human X-linked amelogenesis imperfecta. *Human Molecular Genetics* 19(7)**,** 1230-1247. doi: 10.1093/hmg/ddq001.

Brookes, S.J., Barron, M.J., Smith, C.E.L., Poulter, J.A., Mighell, A.J., Inglehearn, C.F., et al. (2017). Amelogenesis Imperfecta Caused by N-Terminal Enamelin Point Mutations in Mice and Men is driven by Endoplasmic Reticulum Stress. *Hum Mol Genet* 26(10)**,** 1863-1876. doi: 10.1093/hmg/ddx090.

Caterina, J.J., Skobe, Z., Shi, J., Ding, Y., Simmer, J.P., Birkedal-Hansen, H., et al. (2002). Enamelysin (matrix metalloproteinase 20)-deficient mice display an amelogenesis imperfecta phenotype. *J Biol Chem* 277(51)**,** 49598-49604. doi: 10.1074/jbc.M209100200.

Fukumoto, S., Kiba, T., Hall, B., Iehara, N., Nakamura, T., Longenecker, G., et al. (2004). Ameloblastin is a cell adhesion molecule required for maintaining the differentiation state of ameloblasts. *J Cell Biol* 167(5)**,** 973-983. doi: 10.1083/jcb.200409077.

Gibson, C.W., Yuan, Z.A., Hall, B., Longenecker, G., Chen, E., Thyagarajan, T., et al. (2001). Amelogenin-deficient mice display an amelogenesis imperfecta phenotype. *J Biol Chem* 276(34)**,** 31871-31875. doi: 10.1074/jbc.M104624200.

Hu, J.C., Hu, Y., Smith, C.E., McKee, M.D., Wright, J.T., Yamakoshi, Y., et al. (2008). Enamel defects and ameloblast-specific expression in Enam knock-out/lacz knock-in mice. *J Biol Chem* 283(16)**,** 10858-10871. doi: 10.1074/jbc.M710565200.

Hu, Y., Smith, C.E., Richardson, A.S., Bartlett, J.D., Hu, J.C., and Simmer, J.P. (2016). MMP20, KLK4, and MMP20/KLK4 double null mice define roles for matrix proteases during dental enamel formation. *Mol Genet Genomic Med* 4(2)**,** 178-196. doi: 10.1002/mgg3.194.

Hurskainen, T., Moilanen, J., Sormunen, R., Franzke, C.W., Soininen, R., Loeffek, S., et al. (2012). Transmembrane collagen XVII is a novel component of the glomerular filtration barrier. *Cell Tissue Res* 348(3)**,** 579-588. doi: 10.1007/s00441-012-1368-x.

Katsura, K., Horst, J., Chandra, D., Le, T., Nakano, Y., Zhang, Y., et al. (2014). WDR72 models of structure and function: A stage-specific regulator of enamel mineralization. *Matrix Biol*. doi: 10.1016/j.matbio.2014.06.005.

Kuster, J.E., Guarnieri, M.H., Ault, J.G., Flaherty, L., and Swiatek, P.J. (1997). IAP insertion in the murine LamB3 gene results in junctional epidermolysis bullosa. *Mamm Genome* 8(9)**,** 673-681.

Kweon, Y.S., Lee, K.E., Ko, J., Hu, J.C., Simmer, J.P., and Kim, J.W. (2013). Effects of Fam83h overexpression on enamel and dentine formation. *Arch Oral Biol* 58(9)**,** 1148-1154. doi: 10.1016/j.archoralbio.2013.03.001.

Lacruz, R.S., Nakayama, Y., Holcroft, J., Nguyen, V., Somogyi-Ganss, E., Snead, M.L., et al. (2012). Targeted overexpression of amelotin disrupts the microstructure of dental enamel. *PLoS One* 7(4)**,** e35200. doi: 10.1371/journal.pone.0035200.

Masuya, H., Shimizu, K., Sezutsu, H., Sakuraba, Y., Nagano, J., Shimizu, A., et al. (2005). Enamelin (Enam) is essential for amelogenesis: ENU-induced mouse mutants as models for different clinical subtypes of human amelogenesis imperfecta (AI). *Hum Mol Genet* 14(5)**,** 575-583. doi: 10.1093/hmg/ddi054.

Mogi, C., Tobo, M., Tomura, H., Murata, N., He, X.D., Sato, K., et al. (2009). Involvement of proton-sensing TDAG8 in extracellular acidification-induced inhibition of proinflammatory cytokine production in peritoneal macrophages. *J Immunol* 182(5)**,** 3243-3251. doi: 10.4049/jimmunol.0803466.

Mohazab, L., Koivisto, L., Jiang, G., Kytomaki, L., Haapasalo, M., Owen, G.R., et al. (2013). Critical role for alphavbeta6 integrin in enamel biomineralization. *J Cell Sci* 126(Pt 3)**,** 732-744. doi: 10.1242/jcs.112599.

Morasso, M.I., Grinberg, A., Robinson, G., Sargent, T.D., and Mahon, K.A. (1999). Placental failure in mice lacking the homeobox gene Dlx3. *Proc Natl Acad Sci U S A* 96(1)**,** 162-167.

Nakayama, Y., Holcroft, J., and Ganss, B. (2015). Enamel Hypomineralization and Structural Defects in Amelotin-deficient Mice. *J Dent Res* 94(5)**,** 697-705. doi: 10.1177/0022034514566214.

Núñez, S.M., Chun, Y.H., Ganss, B., Hu, Y., Richardson, A.S., Schmitz, J.E., et al. (2016). Maturation stage enamel malformations in Amtn and Klk4 null mice. *Matrix Biol* 52-54**,** 219-233. doi: 10.1016/j.matbio.2015.11.007.

Paine, M.L., Wang, H.J., Luo, W., Krebsbach, P.H., and Snead, M.L. (2003). A transgenic animal model resembling amelogenesis imperfecta related to ameloblastin overexpression. *J Biol Chem* 278(21)**,** 19447-19452. doi: 10.1074/jbc.M300445200.

Parry, D.A., Poulter, J.A., Logan, C.V., Brookes, S.J., Jafri, H., Ferguson, C.H., et al. (2013). Identification of mutations in SLC24A4, encoding a potassium-dependent sodium/calcium exchanger, as a cause of amelogenesis imperfecta. *Am J Hum Genet* 92(2)**,** 307-312. doi: 10.1016/j.ajhg.2013.01.003.

Parry, D.A., Smith, C.E.L., El-Sayed, W., Poulter, J.A., Shore, R.C., Logan, C.V., et al. (2016). Mutations in the pH Sensing G-protein Coupled Receptor GPR68 cause Amelogenesis Imperfecta. *American Journal of Human Genetics* 99(4)**,** 984-990. doi: 10.1016/j.ajhg.2016.08.020.

Ryan, M.C., Lee, K., Miyashita, Y., and Carter, W.G. (1999). Targeted disruption of the LAMA3 gene in mice reveals abnormalities in survival and late stage differentiation of epithelial cells. *J Cell Biol* 145(6)**,** 1309-1323.

Seedorf, H., Klaften, M., Eke, F., Fuchs, H., Seedorf, U., and Hrabe de Angelis, M. (2007). A mutation in the enamelin gene in a mouse model. *J Dent Res* 86(8)**,** 764-768. doi: 10.1177/154405910708600815.

Shin, M., Hu, Y., Tye, C.E., Guan, X., Deagle, C.C., Antone, J.V., et al. (2014). Matrix metalloproteinase-20 over-expression is detrimental to enamel development: a Mus musculus model. *PLoS One* 9(1)**,** e86774. doi: 10.1371/journal.pone.0086774.

Simmer, J.P., Hu, Y., Lertlam, R., Yamakoshi, Y., and Hu, J.C. (2009). Hypomaturation enamel defects in Klk4 knockout/LacZ knockin mice. *J Biol Chem* 284(28)**,** 19110-19121. doi: 10.1074/jbc.M109.013623.

Smith, C.E., Wazen, R., Hu, Y., Zalzal, S.F., Nanci, A., Simmer, J.P., et al. (2009). Consequences for enamel development and mineralization resulting from loss of function of ameloblastin or enamelin. *Eur J Oral Sci* 117(5)**,** 485-497. doi: 10.1111/j.1600-0722.2009.00666.x.

Stephan, A.B., Tobochnik, S., Dibattista, M., Wall, C.M., Reisert, J., and Zhao, H. (2012). The Na(+)/Ca(2+) exchanger NCKX4 governs termination and adaptation of the mammalian olfactory response. *Nat Neurosci* 15(1)**,** 131-137. doi: 10.1038/nn.2943.

Vogel, P., Hansen, G.M., Read, R.W., Vance, R.B., Thiel, M., Liu, J., et al. (2012). Amelogenesis imperfecta and other biomineralization defects in Fam20a and Fam20c null mice. *Vet Pathol* 49(6)**,** 998-1017. doi: 10.1177/0300985812453177.

Wang, S.K., Hu, Y., Yang, J., Smith, C.E., Nunez, S.M., Richardson, A.S., et al. (2015). Critical roles for WDR72 in calcium transport and matrix protein removal during enamel maturation. *Mol Genet Genomic Med* 3(4)**,** 302-319. doi: 10.1002/mgg3.143.

Wang, S.K., Hu, Y., Yang, J., Smith, C.E., Richardson, A.S., Yamakoshi, Y., et al. (2016). Fam83h null mice support a neomorphic mechanism for human ADHCAI. *Mol Genet Genomic Med* 4(1)**,** 46-67. doi: 10.1002/mgg3.178.

Wazen, R.M., Moffatt, P., Zalzal, S.F., Yamada, Y., and Nanci, A. (2009). A mouse model expressing a truncated form of ameloblastin exhibits dental and junctional epithelium defects. *Matrix Biol* 28(5)**,** 292-303. doi: 10.1016/j.matbio.2009.04.004.

Yamakoshi, Y., Richardson, A.S., Nunez, S.M., Yamakoshi, F., Milkovich, R.N., Hu, J.C., et al. (2011). Enamel proteins and proteases in Mmp20 and Klk4 null and double-null mice. *Eur J Oral Sci* 119 Suppl 1**,** 206-216. doi: 10.1111/j.1600-0722.2011.00866.x.
